# A space for time. Exploring temporal regulation of plant development across spatial scales

**DOI:** 10.1111/tpj.70130

**Published:** 2025-03-31

**Authors:** Yadhusankar Sasidharan, Vijayalakshmi Suryavanshi, Margot E. Smit

**Affiliations:** ^1^ Department of Developmental Genetics, Centre for Plant Molecular Biology (ZMBP) Eberhard Karls University Tuebingen D‐72076 Germany

**Keywords:** cell identity regulation, development, Phase transition, temporal regulation, timing

## Abstract

Plants continuously undergo change during their life cycle, experiencing dramatic phase transitions altering plant form, and regulating the assignment and progression of cell fates. The relative timing of developmental events is tightly controlled and involves integration of environmental, spatial, and relative age‐related signals and actors. While plant phase transitions have been studied extensively and many of their regulators have been described, less is known about temporal regulation on a smaller, cell‐level scale. Here, using examples from both plant and animal systems, we outline time‐dependent changes. Looking at systemic scale changes, we discuss the timing of germination, juvenile‐to‐adult transition, flowering, and senescence, together with regeneration timing. Switching to temporal regulation on a cellular level, we discuss several instances from the animal field in which temporal control has been examined extensively at this scale. Then, we switch back to plants and summarize examples where plant cell‐level changes are temporally regulated. As time cannot easily be separated from signaling derived from the environment and tissue context, we next discuss factors that have been implicated in controlling the timing of developmental events, reviewing temperature, photoperiod, nutrient availability, as well as tissue context and mechanical cues on the cellular scale. Afterwards, we provide an overview of mechanisms that have been shown or implicated in the temporal control of development, considering metabolism, division control, mobile signals, epigenetic regulation, and the action of transcription factors. Lastly, we look at remaining questions for the future study of developmental timing in plants and how recent technical advancement can enable these efforts.

## Significance Statement

Developmental timing in plants is tightly regulated at the whole‐organism scale, from germination to flowering. Less is known about when and how local transitions are temporally regulated. Not every cell or tissue change happens at any time, and by now, several examples are known where there appears to be regulation of the timing of these decisions. In this review, we explore examples of developmental transitions whose timing is being controlled and the underlying mechanisms.

## INTRODUCTION

Time is generally associated with change, as Aristotle stated “if nothing changes, there is no time.” This review focuses on how timing is regulated in plant development. While not exhaustive, we aim to provide an overview of currently known examples and mechanisms involved in the temporal control of plant development at various spatial scales. Several excellent recent reviews have previously focused on developmental timing in plants (Coen & Prusinkiewicz, 2024; Swift et al., 2022). In this review, we look mainly at examples and mechanisms of temporal regulation of development in *Arabidopsis thaliana*. We examine questions such as: when are mechanisms in play that slow down or speed up developmental transitions and how do these mechanisms regulate developmental timing?

A first major challenge is defining temporal regulation in the context of development. Temporal control can be imagined as a clock, measuring regular oscillations, or an hourglass, measuring a change in a level until a threshold is reached (Johnson & Day, 2000). Here we mostly focus on the hourglass view, looking less at absolute time and circadian rhythms and instead focusing on the relative timing of developmental events. In plant development, temporal information is often intermingled with spatial and environmental signals; as a result, questions of developmental timing are tightly linked to these factors.

A second challenge is capturing the different scales at which temporal regulation exists. Temporal and environmental signals come together to regulate major phase transitions such as germination and flowering at a systemic level. In addition, recent findings have found that temporal regulation also exists on the scale of a single‐cell type or cell identity. In this review, we will look at mechanisms that act not only on a systemic level, including plant phase transitions, but also at a local, cell environment level.

When we consider the cell and the local scale, we encounter our third challenge: How is a cell's identity defined? When discussing cell identity and fate, we will use terminology similar to that used in Rusnak et al. (2024), where *cell fate* is defined by the ultimate end point of the cell's lineage and *cell identity* changes on the way there. A cell's fate or identity is classically determined by looking at its morphology, location, and function. However, plant cell fate can be flexible and local changes can change their trajectory (Rusnak et al., 2024; Ryu et al., 2021). For example, if leaf epidermal cells undergo abnormal periclinal divisions, inner cells can gain mesophyll fate; furthermore, genetic ablation of root endodermal cells can trigger neighboring pericycle cells to re‐enter the cell cycle to contribute to the loss of ablated cells (Gehrke et al., 2023; Marhavý et al., 2016). In addition to morphology, location, and function, marker lines and transcriptome analyses can help define cell identity. Technological advances such as single‐cell or nucleus approaches and multiplexed *in situ* hybridization help increase the fidelity with which specific cell identities can be defined (Adema et al., 2024; Nobori et al., 2023; Oliva & Lister, 2023). These advances also highlight the identity heterogeneity within a cell type and the challenges in capturing all aspects of a cell's “identity.” In addition, these new features can help to identify transition points between different cell identity stages and confirm the existence of mixed cell identities (Shahan et al., 2022). Here we consider the regulation of cell identity and how identity changes or progression are controlled in time.

In this review, we start by outlining *when* the timing of developmental transitions is regulated. We start with the systemic or global level: describing transitions that involve whole organs or plants. Next, we look at examples of local temporal regulation in animal development, where these processes have been studied more extensively. Then we switch back to plant development to discuss similar local level temporal regulation. Next, instances of *what* factors influence timing are considered, including different systemic and local environmental factors in plants that weigh into determining when transitions take place. Afterwards, we outline *how* temporal control is enacted, considering diverse mechanisms. Finally, we summarize some remaining questions surrounding timing in plant development events and highlight recent technological advances that can contribute to answering these questions. Since our review spans and connects several complex fields, we lack the space to provide extensive background for each field or topic. We therefore refer to excellent in‐depth reviews in the respective field of study indicated with an asterisk (*).

## WHEN: EXAMPLES OF TEMPORAL REGULATION

During the plant's life, its requirements and functionalities change, and it generates dedicated organs and cell types to match these, undergoing large phase transitions as well as more subtle regulatory changes. Historically, temporal control of plant development has primarily been studied at a systemic level. Here, we start by outlining some examples of this systemic level temporal control before discussing examples of temporal control at a local level in both animal and plant systems.

### Plant phase transitions: temporal regulation on a systemic level

**Figure 1:**
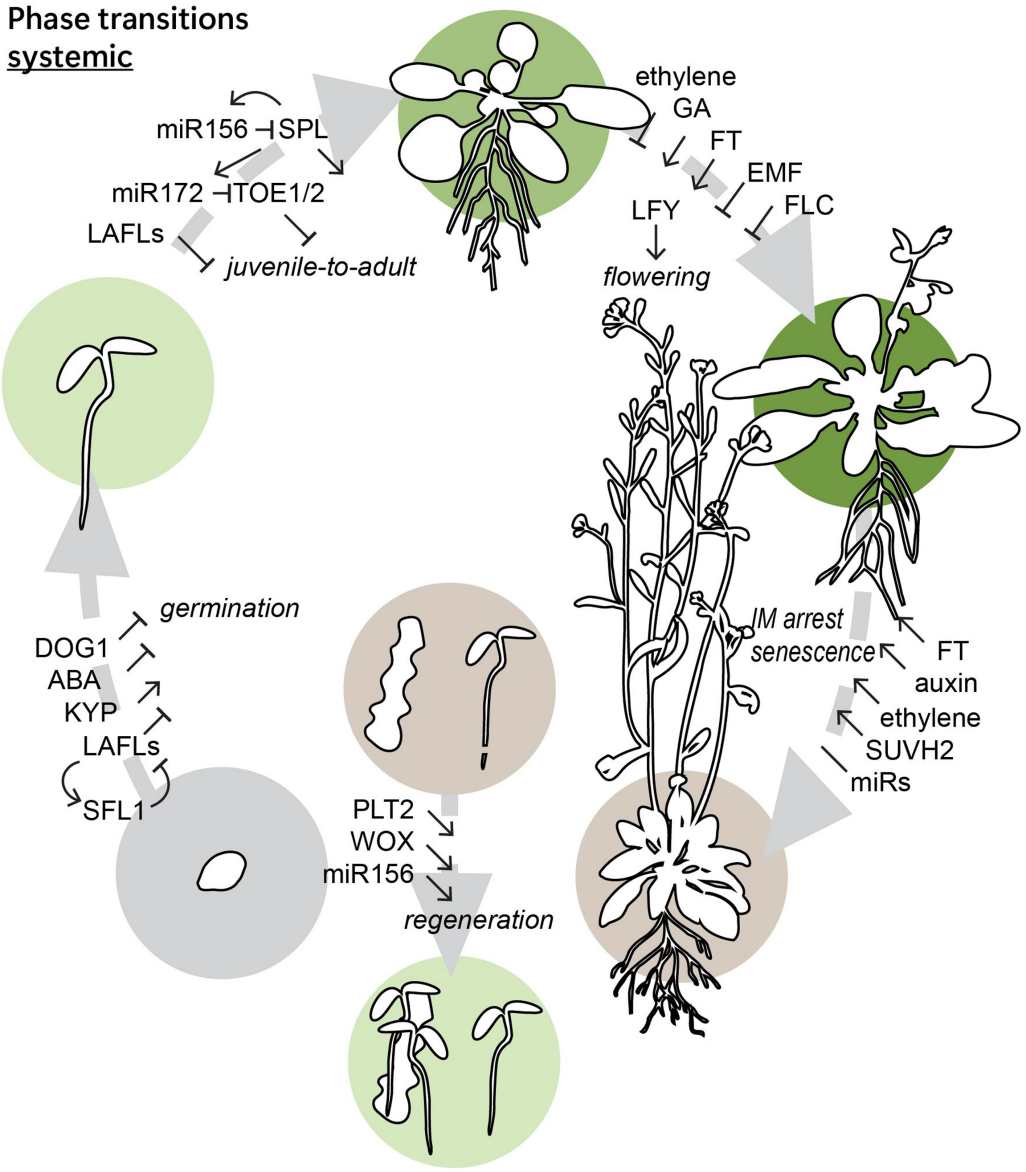
Major plant phase transitions and their regulators. During plant development, the timing of systemic phase transitions (germination, the transition from juvenile to adult, flowering time, and senescence) is regulated by various factors. While not classically included in the phase transitions, upon induction or wounding, regeneration is a similar major reprogramming event which depends on temporal regulation. ABA, abscisic acid; DOG1, delay of germination; EMF, embryonic flower; FLC, flowering locus C; FT, flowering time; FUS3, FUSCA3; IM, inflorescence meristem; KYP, kryptonite; LAFLs, leafy cotyledon1 (LEC1), abscisic acid insensitive3 (ABI3), FUSCA3 (FUS3), and LEC2; LFY, leafy; PLT, plethora; REF, relative of early flowering; SFL, seed dormancy four‐like; SPL, squamosa‐promoter binding protein‐like; SUVH, SU(VAR)3–9 homolog; TOE, target of early activation tagged; WOX, Wuschel‐related homeobox.

Phase transitions mark crucial changes in the plant's body plan, drastically changing its growth and development; as a result, their timing is tightly regulated (Figure 1).

Following seed maturation, seeds initially are dormant until germination is triggered. After ripening, stratification, light, and nitrate all promote germination, with the exact timing of germination depending on the relative strengths of dormancy promoting and breaking factors (Bentsink & Koornneef, 2008; Buijs, 2020; Finkelstein et al., 2008*). The protein DELAY OF GERMINATION (DOG1) and the hormone Abscisic Acid (ABA) accumulate during seed maturation, and help establish dormancy, with dormancy levels varying across Arabidopsis genotypes and correlating with DOG1 and ABA levels (Figure 1) (Bentsink & Koornneef, 2008; Née et al., 2017; Soppe & Bentsink, 2020). In addition, LAFL (LEAFY COTYLEDON1, LEC1, ABSCISIC ACID INSENSITIVE3, ABI3, FUSCA3, FUS3, and LEC2) Transcription factors (TFs) are major regulators of embryogenesis, seed maturation, and dormancy, with LAFL mutants having various dormancy defects, such as *abi3* seeds being viviparous and *fus3* seeds showing reduced dormancy and viability (Gazzarrini et al., 2004; Suzuki et al., 2003). Recent work has expanded on LAFLs preventing precocious germination, identifying interactions between SEED DORMANCY FOUR‐LIKE 1 (SFL1) and its paralogues with LAFLs to influence the timing of dormancy breaking, potentially by affecting chromatin remodeling (Gazzarrini & Song, 2024; Zheng et al., 2022). Other studies further expanded on a role of epigenetic modifications in dormancy, finding that RELATIVE OF EARLY FLOWERING 6 (REF6)‐mediated H3K27 demethylation is necessary for robust germination and that KRYPTONITE (KYP)/SU(VAR)3–9 HOMOLOG (SUVH4), a histone methyltransferase, decreases seed dormancy (Pan et al., 2023; Sajeev et al., 2024; Zheng et al., 2012).

Plants next undergo the juvenile‐to‐adult transition, also called the vegetative phase change, after which leaf morphology changes with adult leaves having a larger length/width ratio and a more complex, serrated shape (Poethig & Fouracre, 2024*). In Arabidopsis under long‐day conditions, this transition occurs after it has produced about six leaves (Doody et al., 2022). On a cellular level, adult leaves have smaller cells and produce trichomes on both surfaces (Manuela & Xu, 2020). The timing of the vegetative phase change is regulated by the miR156/157‐SPL pathway (SQUAMOSA‐PROMOTER BINDING PROTEIN‐LIKE) with miR156/157 levels decreasing over time and SPL protein levels increasing (Figure 1) (Manuela & Xu, 2020; Wu et al., 2009; Zheng et al., 2019). The expression level of miR156/157 forms a threshold and is influenced by environmental signals, epigenetic regulators, and LAFL expression (Gao et al., 2022; He et al., 2018; Wang & Perry, 2013). In addition, LEC2 or FUS3 affects the vegetative phase change, and the loss of either results in a shorter vegetative phase as a result of either increased H3K27me3 or reduced ethylene signaling, respectively (Gao et al., 2022; Gazzarrini & Song, 2024; Lumba et al., 2012).

Next, in the transition to flowering, the morphology and function of the shoot apical meristem are changed to form the inflorescence meristem (Freytes et al., 2021*). Under long‐day conditions, this transition takes place in Arabidopsis after producing 9.5 leaves (Suh et al., 2003). The switch to flowering is preceded by a rapid increase in LEAFY (LFY), a pioneer TF considered the master regulator of flowering (Figure 1) (Jin et al., 2021; Weigel et al., 1992; Weigel & Nilsson, 1995). In short‐day conditions, the floral transition is delayed by several weeks, with LFY expression increasing gradually over time (Blázquez et al., 1997). The timing of the floral transition and LFY expression is regulated by phytohormone (gibberellic acid, ethylene) accumulation and changes in photoperiod signaling which affect histone marks, DNA methylation, TF activity, and FLOWERING LOCUS T (FT) expression (Freytes et al., 2021; Lee et al., 2020; Weigel et al., 1992; Yamaguchi, 2021; Zhu, Chen, et al., 2020). It is crucial that flowering is not induced prematurely and several epigenetic regulators prevent early flowering. EMBRYONIC FLOWER 1 (EMF1) and EMF2 are a transcriptional repressor and a Polycomb group protein that both prevent premature flowering and *emf* mutants flower directly at germination, completely bypassing vegetative shoot growth (Sánchez et al., 2009; Yoshida et al., 2001). Continued expression of flowering repressor FLOWERING LOCUS C (FLC) ensures delayed flowering until after a cold period in winter‐annual Arabidopsis varieties. The FLC expression is controlled by several programs including the activity of FRIGIDA (FRI), epigenetic silencing through VERNALIZATION (VRN) activity, and production of the FLC antisense RNA COOLAIR (Bastow et al., 2004; Maple et al., 2024; Swiezewski et al., 2009).

Finally, at the end of an annual plant's life cycle, it undergoes senescence (Figure 1). Various regulatory TFs, receptors, kinases/phosphatases, phytohormones, epigenetic regulators, and regulatory RNAs act together to set the timing for senescence, including termination of flowering and initiation of leaf senescence (Kim, Kim, et al., 2018). Termination of flowering is highly synchronized, and the inflorescence is arrested through the activity of FLOWERING LOCUS T (FT) at the end of a predetermined though flexible time (González‐Suárez et al., 2020, 2024; Miryeganeh et al., 2018). The timing of floral termination additionally depends on auxin production in recently produced fruits (Ware et al., 2020). The exact timing of leaf senescence is controlled by the integration of ethylene signaling, which promotes senescence (Grbić & Bleecker, 1995); levels of microRNAs miR156, miR164, miR172, and miR840 (Zhang et al., 2021); expression levels of members of the NAC, WRKY, and MYB TF families (Kim, Park, et al., 2018; Woo et al., 2013); and chromatin state affected by SUVH2 levels (Ay et al., 2009). In addition, light affects the timing of senescence, with the ratio of red versus far‐red light (R:FR) affecting senescence through the photoreceptor Phytochrome B (PhyB) and far‐red light via Phytochrome A and the TF FAR‐RED ELONGATED HYPOCOTYL3 (FHY3) (Lee et al., 2021; Sakuraba, 2021; Sakuraba et al., 2014; Tian et al., 2020).

Wounding response, regeneration, and stress‐induced formation of new organs are major reprogramming events that are part of many plants' life cycle (Ikeuchi et al., 2019*; Kareem et al., 2016). The regenerative ability of the plants can be enhanced *in vitro* using different approaches. Several TFs (PLETHORA [PLT], CUP‐SHAPED COTYLEDON, WUSCHEL‐RELATED HOMEOBOX family members), epigenetic modifications, environmental signals, energy status, and mechanical signals have all been shown to modulate regeneration (Figure 1) (Chen et al., 2024; Kim, Yang, et al., 2018; Larriba et al., 2021; Lee et al., 2024; Pan et al., 2019; Serivichyaswat et al., 2022; Shanmukhan et al., 2021). Several of these factors have been shown to affect the temporal dynamics of regeneration. In the root, PLT2/BABYBOOM expression both reflects and influences the different developmental zones, and during root regeneration, it acts in a dose‐dependent manner with increased levels of PLT2 slowing down the root tip re‐establishment (Durgaprasad et al., 2019; Galinha et al., 2007). Shoot regenerative rate and competence from callus are correlated with the level of miR156 (Zhang et al., 2015). Finally, callus of mutants with altered chromatin accessibility develop new shoots faster (increased accessibility) or slower (decreased accessibility) when placed on shoot induction media (Li et al., 2011; Li, Zhang, et al., 2024; Liu, Zhang, et al., 2018; Shemer et al., 2015).

Altogether, the timing of plant phase transitions depends on the integration of many components, with several key players (miR156‐SPL, LAFLs, FT) playing roles in multiple transitions.

### Lessons from animal systems: cell identity

While the timing of plants' major phase transitions has been studied in some detail, our understanding of temporal regulation at a cell identity level is more limited. By contrast, the field of animal development has a more extensive history studying developmental timing at a cellular level. Comparing timing differences between species, investigating temporal morphogen dynamics, and the finding that the specification of certain cell identities depends on developmental timing have all resulted in a robust body of work in temporal regulation in animal development (El‐Danaf et al., 2023; Patil & van Zon, 2024; Wang et al., 2021). Here we look at four examples and use these to think about scales and mechanisms through which the timing of cell development is affected.

**Figure 2:**
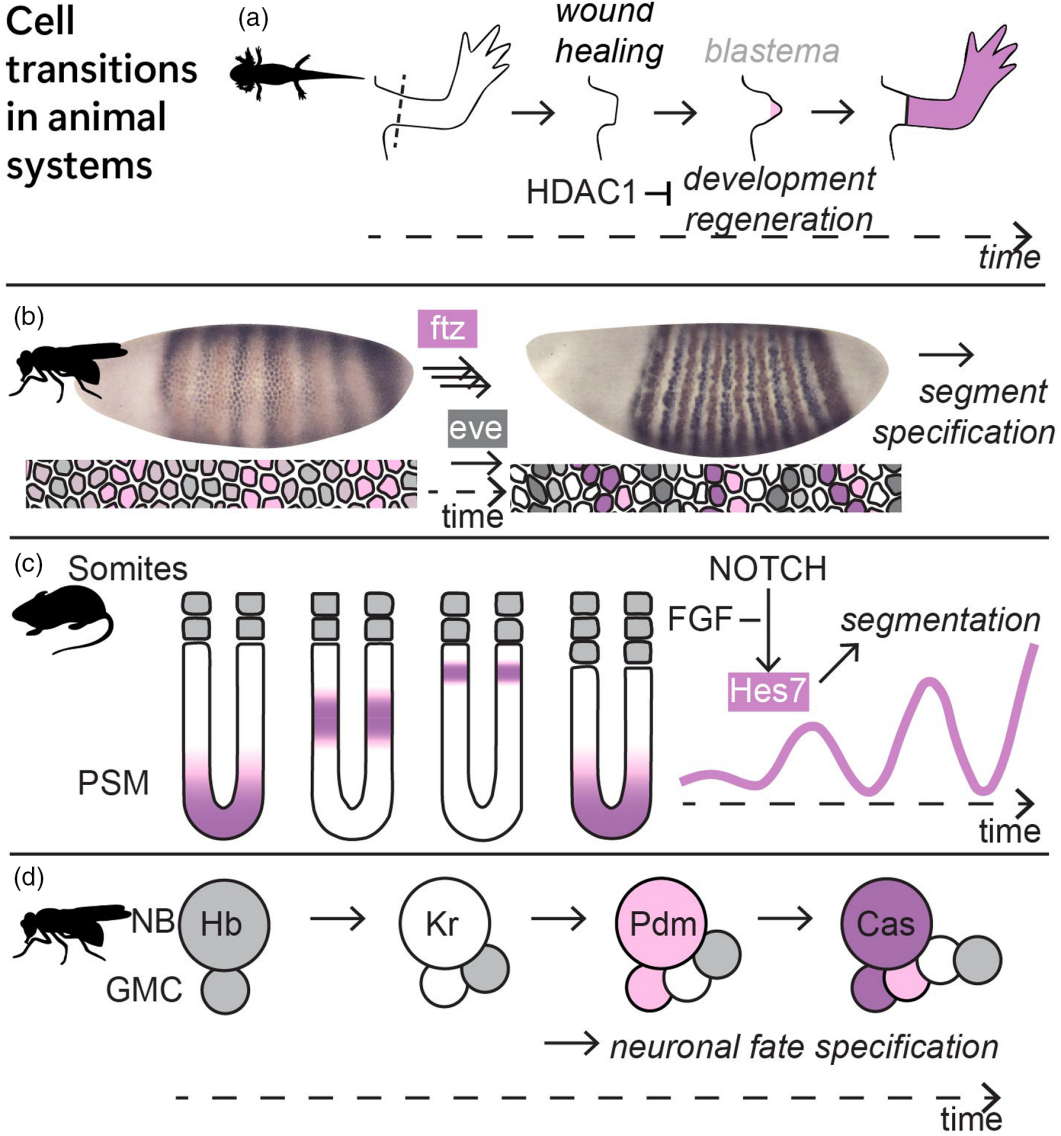
Cell fate transitions in animal systems and their temporal regulators. (a) Axolotl limb regeneration. (b) *Drosophila* larval patterning. (c) Vertebrate presomitic mesoderm segmentation. (d) *Drosophila* neuronal fate specification. Cas, Castor; eve, even‐skipped; FGF, fibroblast growth factor; ftz, fushi tarazu; Hb, Hunchback; HDAC, histone deacetylase; GMC, ganglion mother cell; HES7, Hes family BHLH transcription factor 7; Kr, Kruppel; lin, abnormal cell lineage protein; NB, neuroblast; Pdm1/2, POU domain protein 1 and 2; PSM, presomitic mesoderm.

During Axolotl (*Ambystoma mexicanum*) limb or tail regeneration, the timely remodeling of chromatin prevents premature activation of regeneration and developmental‐related genes (Figure 2a). Single‐cell transcriptomic studies found that differentiated fibroblast cells dedifferentiate and reactivate the embryonic program for limb development (Gerber et al., 2018). Overall, cellular heterogeneity first decreases during blastema formation but increases again as the cells begin to differentiate (Gerber et al., 2018). HISTONE DEACETYLASE (HDAC) inhibiting drugs were found to prevent successful tail regeneration, and HDAC1 expression in nerve cells was shown to be crucial to the regeneration process (Voss et al., 2019; Wang et al., 2019). By unraveling transcriptomic changes during regeneration, researchers have now shown that HDAC1 activity prevents premature upregulation of developmental genes in the wound healing stage (Wang et al., 2021). Thus, here regulation of chromatin accessibility mediates temporal control over cell identity development.

During *Drosophila melanogaster* embryogenesis, the pair‐rule genes *fushi tarazu* (*ftz*) and *even‐skipped* (*eve*) undergo dynamic shifts in their expression before defining the edges of individual segments (Figure 2b) (Lim et al., 2018). Live imaging revealed that *ftz* stripes move faster than *eve* stripes (Lim et al., 2018). Transcription changes from short, dim bursts to longer, brighter bursts over time and depends on the activities of two enhancer elements (Birnie et al., 2023). Next, patterning of the anteroposterior axis is controlled by sequential expression of three timer genes: *caudal*, *dichaete*, and *odd‐paired* (Clark et al., 2022). In the tail region, their expression is delayed, and this region segments later, after blastocyst segmentation (Clark et al., 2022). In short, the speed and relative timing of patterning factors help with correct and timely fate assigning.

Temporal oscillations of pair‐rule genes generate repetitive epithelial blocks during somitogenesis in arthropods and vertebrates (Figure 2c) (Clark et al., 2019). The timing of segmentation‐related oscillations differs between species and can have dramatic effects on the final body plan: faster segmentation in corn snake embryos results in the formation of many more, smaller somites as compared to zebrafish, mouse, and chicken embryos that share conserved gradient systems (Gomez et al., 2008). When comparing mouse and human systems (2–3 versus 5–6 h oscillations), the speed of biochemical reactions including protein production and degradation was found to contribute to the difference in developmental rate in the embryos (Diaz‐Cuadros et al., 2023; Matsuda et al., 2020). In addition, the number of introns in the mouse *Hes Family BHLH Transcription Factor 7* (*Hes7*) gene contributes to segmentation timing: when two *Hes7* introns are removed, its oscillation and somite segmentation are sped up by about 9% (Harima et al., 2013). Finally, in cultured zebrafish presomitic mesoderm (PSM) cells, exogenous signals such as FIBROBLAST GROWTH FACTOR are not required to generate a wave pattern, but they can slow down this internal timer *in vitro* (Rohde et al., 2024). Thus, differences in segmentation timing are essential, and they are mediated by basic properties of the factors involved.

Finally, during drosophila neuroblast (NB) development, spatial information initially determines neuroblast identity, but the subsequent specification of ganglion mother cell (GMC) fates takes place without further spatial information (Figure 2d). Instead, NBs undergo several rounds of asymmetric divisions, each time producing a large NB and a small GMC that inherits the expression of the current fate‐regulating temporal TF (Doe, 2017; Pollington et al., 2023). NBs and the formed GMCs subsequently express Hunchback (Hb), Kruppel (Kr), POU domain protein 1 and 2 (Pdm1/2), and Castor (Cas) to regulate unique neuronal fates (Grosskortenhaus et al., 2005; Homem & Knoblich, 2012). In models, general activation with feedback repression appears sufficient to generate the temporal TF cascade, with the first transition (Hb to Kr) depending on cell division (Doe, 2017; Grosskortenhaus et al., 2005). This is an example of cell identity specification depending on the timing of its specification through division.

In animal development, the regulation of cell identity and pattern formation at a local level is known to involve temporal in addition to spatial mechanisms. Drawing inspiration from this field, we are interested in exploring similar mechanisms in plant development (Meyerowitz, 2002). To study the temporal regulation of local development in plants, the identification of transitions whose timing is precisely regulated remains the main challenge.

### Cell identity over time: temporal regulation on a local level

A cell's identity is tightly regulated and generally progresses toward its final cell fate along a set trajectory. Many studies have sought to elucidate regulators of cell fate and identity, but few have identified temporal regulators of identity progression. In this section, we explore examples of local regulation of developmental timing, considering single cell and organ level control.

**Figure 3:**
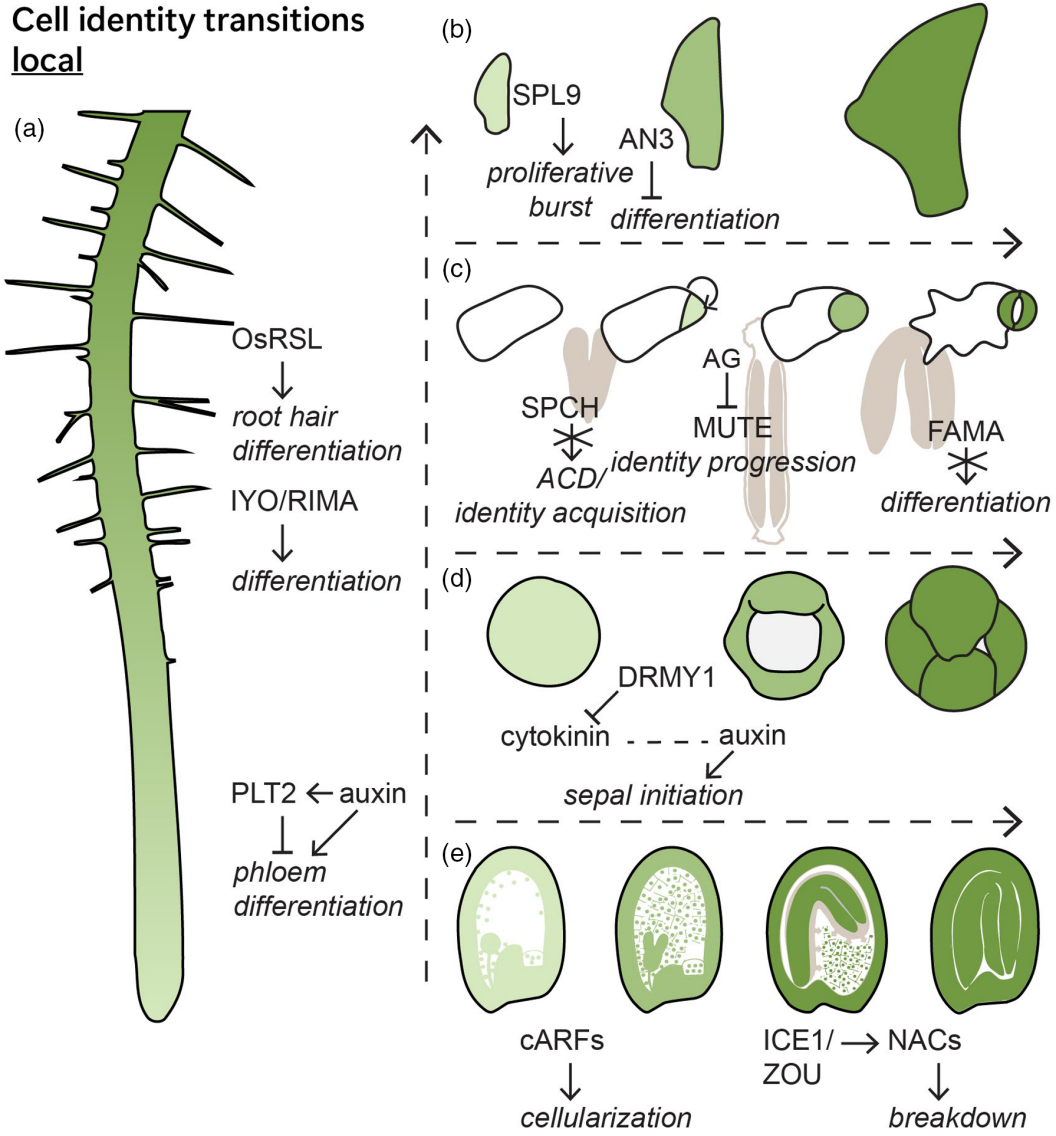
Plant cell identity transitions regulators. (a) Cell differentiation in root development. (b) Leaf cell proliferation and expansion. (c) Stomatal lineage progression during embryogenesis and gynoecial valve development. (d) Endosperm cellularization and breakdown. (e) Sepal initiation robustness and uniformity. ACD, asymmetric cell divisions; AG, agamous; AN, angustifolia 3/GRF‐interacting factor; cARF, clustered auxin response factor; DRMY, development‐related MYB‐like; ICE, inducer of CBP expression; IYO, miniyo; NAC, NAM ATAF CUC; OsRSL, RHD six‐like; PLT, plethora; SPCH, speechless; SPL, squamosa‐promoter binding protein‐like.

In the root, space and time are aligned along the root's length (Motte et al., 2019*). This makes it relatively straightforward to track development but challenging to separate spatial from temporal regulation. In general, the sizes of different root zones correlate with the time spent in each zone and mutants affected in their root zonation often also have “faster” or “slower” cell identity development when measured spatially (Verbelen et al., 2006). Factors such as the PLTs, which affect root zone sizes, appear to have very general roles that include both spatial and temporal effects (Aida et al., 2004). However, general regulators of development and differentiation such as MINIYO (IYO) and RPAP2 IYO Mate (RIMA) were shown to speed up or delay differentiation events across root cell types (Figure 3a) (Muñoz et al., 2017; Sanmartín et al., 2011). Root cell identities strongly influence each other, and ablation experiments have shown a strong influence of a cell's position on its fate (Gehrke et al., 2023; van den Berg et al., 1995). However, development of some root cell identities appears controlled in a temporal manner: PLT2 misexpression can delay phloem differentiation while the PAX–BRX–PIP5K1 module (PROTEIN KINASE ASSOCIATED WITH BRX, BREVIS RADIX and PHOSPHATIDYLINOSITOL‐4‐PHOSPHATE‐5‐KINASE) mediated auxin canalization speeds up differentiation (Aliaga Fandino et al., 2024; Roszak et al., 2021). In rice, higher order OsRSL (RHD SIX‐LIKE) mutants have delayed root hair emergence while misexpression of OsRSLs results specifically in premature root hair differentiation (Kim et al., 2017).

During leaf development (Lv et al., 2023*), a proliferative phase is followed by differentiation progressing from tip to base. In juvenile leaves, a proliferative burst takes place, correlating with SPL9 levels (Figure 3b) (Li, Jenke, et al., 2024). In addition, ANGUSTIFOLIA 3/GRF‐INTERACTING FACTOR 3 (AN3), a positive regulator of cell proliferation, was shown to prevent precocious differentiation during leaf development (Ezaki et al., 2024).

In the stomatal lineage, identity transitions are mediated by master regulator bHLH TFs (Bergmann & Sack, 2007*; Kim et al., 2022; Liu, Mair, et al., 2024; Smit & Bergmann, 2023). While these regulators are generally considered necessary and sufficient, recent discoveries suggest that this is not always the case. SPEECHLESS (SPCH) plays a dominant role in promoting Asymmetric Cell Divisions (ACDs) and lineage entry but does not and can not induce this change during the heart to torpedo stages of embryogenesis (Figure 3c) (MacAlister et al., 2007; Smit et al., 2023). After lineage initiation, the timing of MUTE expression and Guard Mother Cell (GMC) identity are influenced by a cell size threshold, and this change is in turn accompanied by lengthening of the cell cycle as the cell proceeds to divide symmetrically (Gong et al., 2023; Han et al., 2022; Pillitteri et al., 2007). This transition is delayed in stomatal cells located on the gynoecial valves, where stomatal cells are arrested as meristemoids through the action of the floral regulator AGAMOUS (AG) (Brazel et al., 2023). AG represses *MUTE* transcription, which ensures that stomatal maturation coincides with fertilization (Brazel et al., 2023). Finally, FAMA's role driving guard cell differentiation is hampered during embryogenesis, a time when stomatal cells do not but apparently also can not differentiate (Ohashi‐Ito & Bergmann, 2006; Smit et al., 2023). This temporal block can be circumvented by removing embryos from the ovule, suggesting a role for extraembryonic factors (Zuch et al., 2023).

Robust timing of sepal initiation is needed for proper flower bud closure (Roeder, 2021*) (Figure 3d). Mutants with altered sepal initiation timing have varying sepal sizes resulting in incomplete flower bud closure (Zhu, Klasfeld, et al., 2020). Flower bud closure is influenced by DEVELOPMENT RELATED MYB‐LIKE1 (DRMY1) which regulated TARGET OF RAPAMYCIN (TOR) activity, ribosomal content, and translation, eventually controlling the timing and position of sepal initiation inhibition (Kong, Zhu, Pan, et al., 2024; Kong, Zhu, Scarpin, et al., 2024).

Finally, the timing of endosperm cellularization and subsequent elimination is tightly controlled and affects seed size and viability (Figure 3e) (Bente & Köhler, 2024; Doll & Ingram, 2022*; Hehenberger et al., 2012). The timing of cellularization is controlled through the expression of clustered AUXIN RESPONSE FACTOR (cARF) with levels of cARF depending on parental contributions: later expression results in a delay and earlier expression in premature cellularization (Butel et al., 2024). As the embryo expands, the endosperm needs to be eliminated to make space. Programmed cell death (PCD) of endosperm cells needs to happen slowly enough to keep supporting the embryo but quickly enough to prevent restriction of embryo growth. ZHOUPI and INDUCER OF CBP EXPRESSION 1 (ICE1) are two TFs that are required for this endosperm breakdown through their control of downstream NACs that promote Programmed Cell Death (Denay et al., 2014; Doll et al., 2023).

Overall, while developmental timing has not been a major focus in studies of cell identity and patterning, above examples indicate that temporal control is also present on a local level, often in specific organ contexts.

## WHAT: FACTORS THAT INFLUENCE TEMPORAL REGULATION

Plants need to adapt their body plan to the environment, responding to changes in factors such as temperature, light conditions, salinity, and nutrient availability. In addition to the environmental conditions sensed by the plant, each cell also responds to its direct environment. Here we describe several environmental factors that were described to affect the timing of developmental events.

### Systemic environment

Temperature affects plant form either directly through temperature‐dependent action of regulatory factors or indirectly by affecting metabolism and water homeostasis (Li et al., 2022*). Here we focus on how temperature directly influences the regulation of major transitions, the main example being seasonal temperature regulating the timing of flowering. To prevent premature flowering, the cold‐activated bHLH TF ICE1 induces expression of *FLC*, resulting in SUPPRESSOR OF OVEREXPRESSION OF CO 1 (SOC1) repression and delayed flowering (Lee et al., 2015). By contrast, at optimal temperatures, SOC1 reduces ICE1 binding to the FLC locus (Lee et al., 2015). This results in a feedback loop integrating cold with other floral signals to ensure optimal timing. By contrast, high temperatures speed up the transition to flowering. Both PHYTOCHROME INTERACTING FACTOR 4 (PIF4) and the alternative splicing of FLOWERING LOCUS M (FLM) are temperature‐responsive and act upstream of FLOWERING LOCUS T (FT) to speed up flowering under warmer temperatures (Balasubramanian et al., 2006; Jin & Ahn, 2021). Winter cold is a required signal for the flowering of winter‐annual varieties of Arabidopsis. Vernalization activates the expression of VRN genes (VRN1, VIN3, and VIN5) that contribute to the silencing of the floral repressor FLC, thereby inducing flowering (Maple et al., 2024). Separately, FRIGIDA (FRI) forms nuclear condensates under cold temperatures, taking it away from the *FLC* locus where it promotes H3 trimethylation at K4 and K36 and transcriptional activation under warm conditions (Zhang et al., 2023). The antisense RNA *COOLAIR* can physically associate with the FLC locus and accelerates its shutdown in the cold by promoting FRI condensates (Csorba et al., 2014; Zhu et al., 2021). Temperature also affects the timing of germination. Germination at low temperatures is slowed down by the cold‐induced accumulation of DOG1. By contrast, seed dormancy can be released via stratification, where cold temperature and dark result in changes in the expression of ABA and GA‐related genes (Yan & Chen, 2020).

Other major environmental factors regulating plant phase transitions include light and photoperiod (Li et al., 2022*). Phytochromes, cryptochromes, and phototropins sense light and control, among others, the timing of senescence and flowering (Lymperopoulos et al., 2018). For instance, the ratio of red versus far‐red light (R:FR) affects the timing of leaf senescence via PhyB and FHY3 (Lee et al., 2021; Sakuraba, 2021; Sakuraba et al., 2014; Tian et al., 2020). FHY3 competes with PIF4 (PHYTOCHROME INTERACTING FACTOR) to repress NON YELLOWING1 and STAY GREEN1 (NYE1 and SGR1), two key regulators of leaf senescence, leading to delayed senescence (Wang et al., 2023). The B BOX (BBX) family of TFs contributes to light‐regulated developmental processes including flowering (Song et al., 2020). BBX13 acts as a negative regulator by delaying flowering under long‐day conditions by affecting CO binding on the *FT* promoter (Rahul et al., 2024).

Salinity and nutrient availability were found to affect the speed of germination and the floral transition. For example, under fluctuating salinity conditions and during high osmotic stress levels, ERECTA (ER) modulates seed germination, and reduced ER signaling under these conditions lowers and slows down germination (Nanda et al., 2019). ABA is likely a mediator of this process as ABA‐related genes are upregulated under salinity stress, and ER triple mutants showed increased ABA sensitivity (Nanda et al., 2019). Flowering time partially depends on nutrient availability: Under low N conditions, the phosphorylation of FLOWERING BHLH 4 (FBH4) is reduced, resulting in nuclear localization of FBH4 and activating CO/FT pathways promoting early flowering (Sanagi et al., 2021). Under low P conditions, Arabidopsis flowering is delayed through the reduction of FT, LFY, and APETALA1 transcripts (Cho et al., 2017). Conversely, increased P did not speed up flowering, indicating that the nutrients themselves might not supply specific signals, but instead, limiting conditions slow down development.

### Local environment

Each cell experiences its own micro‐environment with local cues influencing cell growth and identity. A cell can adopt a new identity depending on its neighbors as shown in cell ablation experiments (Gehrke et al., 2023; van den Berg et al., 1995). Mobile signals such as hormones, peptides, and microRNAs (miRNAs) inform a cell of its environment and guide identity changes over time (see the How section). For example, CLE33 and CLE45, as well as auxin levels, both control the timing of protophloem sieve element differentiation (Aliaga Fandino et al., 2024; Carbonnel et al., 2023).

In addition to mobile signals, a cell's exposure to mechanical cues can influence its development. In response to wounding, cell ablation, or stretching experiments, mechanical signals influence cell division orientation and gene expression, indirectly or directly affecting cell identity (Hamant et al., 2008; Majda et al., 2022; Roszak et al., 2021; Zhang et al., 2022). In addition, mechanical conflicts can be created through alterations in cell wall properties, resulting in local heterogeneity or overall changed properties, eventually altering cell polarity and/or identity (Varapparambath et al., 2022; Wolf, 2022). However, there are currently no examples known of mechanical cues and cell wall properties regulating development timing for.

## HOW: MECHANISMS OF TEMPORAL REGULATION

After exploring examples of the regulation of development timing, here we discuss mechanisms employed to regulate developmental timing. In addition, often the outputs of several mechanisms are integrated, increasing complexity and robustness.

### Metabolic rates and protein turnover

**Figure 4:**
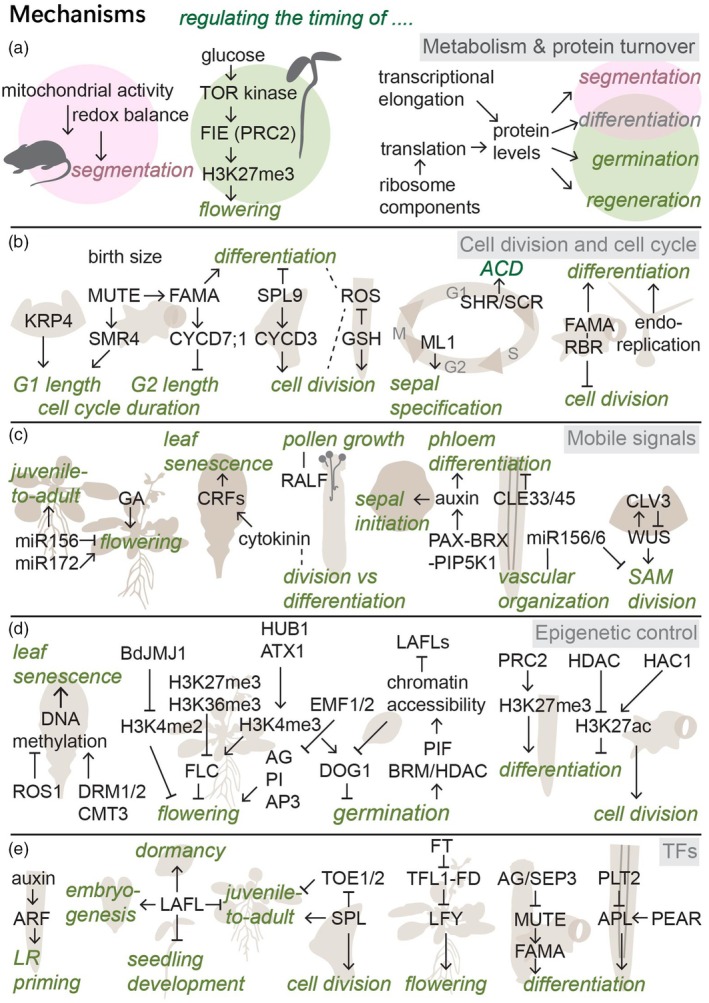
Examples of mechanisms regulating the timing of plant developmental events. (a) Metabolism and turnover affecting animal and plant development. (b) Regulation of cell division, cell cycle duration, and progression. (c) Mobile signals: hormones, peptides, and miRNAs. (d) Epigenetic modifications and chromatin accessibility. (e) TFs. ACD, asymmetric cell divisions; AG, agamous; AN, angustifolia 3/GRF‐interacting factor; AP, apetala; APL, altered phloem development; ARF, auxin response factor; ATX, Arabidopsis trithorax; BdJMJ, jumonji; BRM, brahma; CLE33, clavata3/embryo surrounding region‐related33; CMT, chromomethylase; CRF, cytokinin response factor; CUC, cup shaped cotyledon; CYCD, cyclin D; DOG1, delay of germination; DRM, dormancy‐associated protein; EMF, embryonic flower; FIE, fertilization‐independent endosperm; FLC, flowering locus C; FD, flowering locus D; FT, flowering time; GA, gibberellic acid; GSH, glutathione; HAC, histone acetyltransferase; HDAC, histone deacetylase; HUB, histone monoubiquitination; KRP, KIP‐related protein; KYP, kryptonite; LAFLs, leafy cotyledon1 (LEC1), abscisic acid insensitive3 (ABI3), FUSCA3 (FUS3), and LEC2; LFY, LEAFY; ML, meristem layer; PAX–BRX–PIP5K1, protein kinase associated with BRX, brevis radix, and phosphatidylinositol‐4‐phosphate‐5‐kinase; PEAR, phloem early DNA‐binding‐with‐one‐finger; PI, pistillata; PIF, phytochrome interacting factor; PLT, plethora; PRC, polycomb repressive complex; RALF, rapid alkalinization factor; RBR, retinoblastoma‐related; REF, relative of early flowering; ROS, reactive oxygen species; SCR, scarecrow; SEP, sepalata; SFL, seed dormancy four‐like; SHR, shortroot; SMR, siamese‐related; SPL, squamosa‐promoter binding protein‐like; TFL, terminal flower; TOE, target of early activation tagged; TOR, target of rapamycin; WOX, Wuschel‐related homeobox.

Metabolic rate and protein turnover set the pace for rates of growth and development. In animals, metabolic rate scales with body size and is shown to influence cell fate (Diaz‐Cuadros et al., 2023; Kleiber, 1947). In animal systems, NAD(H) redox balance and mitochondrial activity, respectively, can partially explain species differences in the speed of the segmentation clock and in the rate of neuronal maturation (Figure 4a) (Diaz‐Cuadros et al., 2023; Iwata et al., 2023; Rayon, 2023). In plants, metabolism can affect development through glucose signaling, with glucose‐activated TOR kinase influencing genome‐wide H3K27me3 through the phosphorylation of FERTILIZATION‐INDEPENDENT ENDOSPERM (FIE), a component of the Polycomb Repressive Complex 2 (PRC2) complex (Ye et al., 2022). Inhibiting TOR kinase activity results in a decrease in overall H3K27me3 levels and a delay in the transition to flowering (Ye et al., 2022).

RNA and protein metabolism have been implicated in controlling the timing of developmental transitions and differentiation. The rate of transcriptional elongation is correlated with the ability to differentiate in both animal and plant systems (Figure 4a) (Li et al., 2021; Sanmartín et al., 2012). In Arabidopsis, IYO, an interactor of RNA polymerase II, together with its interactor RIMA influences the timing of differentiation, and misexpression of either results in premature differentiation across cell types (Muñoz et al., 2017; Sanmartín et al., 2011). In addition, an imbalance in ribosome components affects translation of developmental regulators, and abnormal translation dynamics result in the production of ectopic non‐hair cells in the root, slower tissue regeneration, and slower seed germination (Horiguchi et al., 2012; Wang et al., 2020). Finally, protein degradation rates affect the speed of the segmentation clock in human and mouse systems: altered Hes7 protein stability results in altered cell cycle length and segmentation (Matsuda et al., 2020; Rayon et al., 2020).

### Cell division and cell cycle‐dependent regulation

Temporal regulation of cell identity changes often involves coordination of cell cycle length and division dynamics. Cell cycle length varies across organisms, cell types, and developmental stages, with various mechanisms responsible for its regulation (Jones et al., 2019; Loeffler, 2023; Soufi & Dalton, 2016). Sizer‐based mechanisms have been described to control division timing in plant SAM and stomatal cells. In the SAM, cell cycle progression depends on KIP‐related protein 4 (KRP4) levels, and KRP4 gets diluted by cell growth to time the G1/S transition (Figure 4b) (D'Ario et al., 2021). In stomatal development, meristemoids undergoing ACDs change to GMC fate when a birth size threshold is reached (Gong et al., 2023). Upon transition to GMC fate, MUTE then induces the expression of SIAMESE‐RELATED (SMR) proteins including SMR4, lengthening G1, resulting in a slower Symmetric Cell Division (SCD) (Han et al., 2022). Thus, in this case cell cycle length and timing, both seem to reflect and affect cell identity. In general, in leaf, relative organ age affects division rate and cell cycle length: leaf number and cell position along the leaf's proximodistal axis affect SPL9 levels, which promote division by activating CYCLIN D3 family genes (Li, Zhang, et al., 2024). As a result, younger leaves undergo a proliferative burst with faster cell cycles and a delay in differentiation and maturation (Li, Jenke, et al., 2024). In the root, cells instead speed up their cell cycle as they age. This change in division rate is affected by reactive oxygen species (ROS), and recent findings show that the antioxidant glutathione enables fast divisions during regeneration by causing a truncated G1 phase in cells that reprogram their fate first (Lee et al., 2025; Rahni & Birnbaum, 2019; Tsukagoshi et al., 2010).

### Mobile signals

Mobile signals are crucial for coordinating development by integrating local positional information, but developmental timing is also influenced by them. Here we discuss how hormones, peptides, and microRNAs contribute to the regulation of developmental transitions.

Hormone levels and signaling define thresholds for several phase transitions (Figure 4c). For example, GA levels control the timing of flowering, and reduced GA levels delay flowering (Lee et al., 2020). Cytokinins influence the leaf senescence timing through the activity of cytokinin‐induced CYTOKININ RESPONSE FACTOR (CRF) TFs (Wu et al., 2021). As a result, *crf1,3,5,6* mutants have delayed leaf senescence, while CRF1/3/5‐overexpressing leaves undergo senescence earlier (Raines et al., 2016). Hormone dynamics also influence local developmental events. Cytokinins speed up cell division, delay the onset of differentiation, and extend the period of cell proliferation in leaves (Skalák et al., 2019; Wu et al., 2021; Zhang et al., 2005). During gynoecium development, cytokinin levels similarly control the balance between division and differentiation in internal tissues and outgrowths (Cerbantez‐Bueno et al., 2024). Here, cytokinin signaling and levels of D3‐type cyclins determining cell cycle timing are connected by the TF SPATULA (SPT) (Cerbantez‐Bueno et al., 2024). Auxin affects the timing of protophloem sieve element differentiation (Aliaga Fandino & Hardtke, 2022). Phloem sieve element cells differentiate before other cell types in the root; the timing of this differentiation is regulated by the polarly localized PAX–BRX–PIP5K1 module, which controls local auxin canalization, speeding up differentiation (Aliaga Fandino et al., 2024). When instead this module is assembled ectopically in developing xylem vessels, it promotes faster differentiation of xylem vessels (Aliaga Fandino et al., 2024). Looking at combined hormone effects, auxin‐cytokinin dynamics also regulate spatiotemporal sepal patterning, and disrupting either affects the relative timing of sepal initiation, and as a result the number and size of sepals (Kong, Zhu, Pan, et al., 2024; Kong, Zhu, Scarpin, et al., 2024).

Plants produce and respond to signaling peptides and small proteins to guide development; here, we use FT, CLAVATA3/EMBRYO SURROUNDING REGION‐RELATED (CLE), and Rapid ALkalinization Factor (RALF) peptides as examples in temporal regulation at systemic and local levels. *FT* encodes a small mobile protein whose transcription in the phloem depends on environmental factors. After production in the leaf phloem, the protein acts in the SAM together with FLOWERING LOCUS D (FD) to control the timing of the transition to floral fate (Corbesier et al., 2007; Freytes et al., 2021). CLE peptide signaling regulates proliferation and differentiation rates in both apical and vascular meristems (Berckmans et al., 2020; Etchells & Turner, 2010; Willoughby & Nimchuk, 2021). The most studied, CLAVATA3 (CLV3), forms part of a feedback loop with WUSCHEL (WUS) controlling SAM size (Figure 4c) (Han et al., 2020). During embryogenesis, removing CLV3 results in delayed downregulation of WUS, continued division, and an eventually larger WUS domain (Schoof et al., 2000). In the vasculature, CLE33 and 45 prevent neighboring cells from acquiring protophloem identity and prevent young protophloem cells from differentiating prematurely (Carbonnel et al., 2023). Several RALF family members were shown to control and be controlled during pollen‐pistil interactions involving spatial and temporal regulation by the two parents (Abarca et al., 2021). These RALFs control when pollen tube rupture takes place and whether, later, a second pollen tube can exit the septum if fertilization fails (Somoza et al., 2021; Zhong et al., 2022).

Finally, mobile miRNAs regulate spatial and temporal development in the vegetative phase transition, flowering, and vascular patterning among others (D'Ario et al., 2017; Liu, Yu, et al., 2018). miR156 and miR172 play pivotal roles in the juvenile‐to‐adult transition by regulating SPL and AP2‐like transcripts (Figure 4c) (He et al., 2018; Poethig & Fouracre, 2024). Overexpression of miR156 prolongs the vegetative phase and delays flowering while overexpression of miR172 accelerates flowering; however, the exact mechanisms and factors controlling miR156 and miR172 levels and the timing of the transition remain unresolved (Poethig & Fouracre, 2024; Wu et al., 2009). miR399 and miR169 both induce flowering in response to environmental signals: miR399 is temperature‐responsive and targets the phosphate homeostasis gene PHOSPHATE 2 (PHO2), and miR169 is upregulated under abiotic stress to target AtNF‐YA2 transcripts (Liu, Yu, et al., 2018). Finally, miR165 and miR166 target PHABULOSA and REVOLUTA and play central roles in vascular development and during embryogenesis; their timely sequestration by ARGONAUTE 10 is required for SAM development and maintenance (Miyashima et al., 2011; Zhou et al., 2015).

### Epigenetic control

Epigenetic modifications and resulting changes in chromatin accessibility often correlate with cell identity changes (Birnbaum & Roudier, 2017; Huang & Sun, 2022; Ma et al., 2019; She et al., 2013). Here, we discuss the roles of epigenetic mechanisms such as DNA methylation and histone tail modifications in controlling the timing of developmental transitions.

DNA methylation of promoter regions is influenced by and influences development through gene repression (Hemenway & Gehring, 2023). DNA methylation declines during senescence and influences the timing of the onset of leaf senescence, with hypermethylated REPRESSOR OF SILENCING1 (*ros1*) and hypomethylated triple *DORMANCY‐ASSOCIATED PROTEIN 1/2*, *CHROMOMETHYLASE 3* (drm1/2 cmt3, ddc) mutants displaying a fast or slower onset of leaf senescence, respectively (Figure 4d) (Ogneva et al., 2016; Vatov et al., 2022). However, both types of mutants show a faster progression after the first symptoms, and the underlying mechanisms remain unknown (Vatov et al., 2022). The timing of flowering is also influenced by DNA methylation, which eventually affects the FLC expression. The quadruple *drdd* (DEMETER (DME) family of 5‐methylcytosine DNA glycosylases) mutants show early flowering due to hypermethylation and reduced FLC expression at the seedling stage (Vatov et al., 2022; Williams et al., 2022). On the local scale, DNA methylation‐free plants have severe developmental defects (He et al., 2022).

Modification of histone tails alters chromatin accessibility and influences gene expression levels. Flowering time is one of the developmental transitions heavily regulated by histone 3 modifications. In winter‐annual Arabidopsis varieties, FLC transcription is upregulated by H3K4me3 deposition promoted by HISTONE MONOUBIQUITINATION1 (HUB1) and ARABIDOPSIS TRITHORAX1 (ATX1) (Figure 4d) (Lu et al., 2024). In addition, vernalization promotes silencing of the floral repressor FLC by replacing active H3K36me3 with repressive H3K27 trimethylation (Huang et al., 2021). In *Brachypodium distachyon*, the timing of flowering is influenced by the histone demethylase JUMONJI1, which is required for H3K4me2 demethylation at the loci of flowering time regulations VRN1 and INDETERMINATE1 (Liu, Li, et al., 2024). During seed maturation, H3K4 methyltransferases (ATXs), along with HUB genes and the proteasome alpha subunit F1 (PAF1) complex, promote DOG1 transcription to induce dormancy and regulate germination timing (Xiao et al., 2017). Upon germination, large changes in chromatin accessibility result in the repression of LAFLs and DOG1 and an increased GA/ABA ratio, necessary for the transition to postembryonic growth (Xiao et al., 2017). This chromatin reorganization is in part facilitated by PHYTOCHROME INTERACTING FACTORS (PIFs) upon exposure to light, together with chromatin regulators BRAHMA (BRM) and HDACs, resulting in large transcriptomic changes upon germination (Bouyer et al., 2017; Kawakatsu et al., 2017; Liew et al., 2024; Xiao et al., 2017).

EMF1 and EMF2 contribute to the regulation of histone tail methylation and ubiquitination to regulate flowering, and removing either results in precocious flowering upon germination (Yoshida et al., 2001). EMF1 is a member of the PRC1 complex, and EMF2 is part of the EMF‐PRC2 complex, and they act to repress the flower homeotic genes *AGAMOUS* (*AG*), PISTILLATA, and APETALA3 (Kim et al., 2010; Yang et al., 2017).

On a more local level, in root development, HDAC activity promotes differentiation and the PRC2 complex contributes to maintaining the cell's differentiated status (Ikeuchi, Iwase, Rymen, et al., 2015; Ikeuchi, Iwase, & Sugimoto, 2015). Advances in single cell/nucleus ATACseq approaches are now allowing more in‐depth investigations of how changes in chromatin accessibility correlate with and potentially result in changes in cell identity (Dorrity et al., 2021; Shahan et al., 2021). For example, in the stomatal lineage, reprogramming of accessibility occurs at the transition to differentiation, and the SWI/SNF complex and HISTONE ACETYLTRANSFERASE1 (HAC1) are required to maintain the differentiated, non‐dividing state of mature Guard Cells (Kim et al., 2022; Liu, Mair, et al., 2024). Thus, epigenetic changes can control the timing of phase transitions and are correlated with cell identity changes.

### Transcription factors and gene regulatory networks

During plant development, cell identity and fate are regulated by complex gene regulatory networks involving various Transcription Factors (TFs). The structures of these allow for the integration of various signals and generation of temporal variation in target gene expression, resulting in expression pulses or delays (Alvarez et al., 2021; Coen & Prusinkiewicz, 2024; Van Den Broeck et al., 2020). Together with space‐ and time‐specific expression of regulators, these structures ensure that cell fate and identity changes are robustly controlled. Here we discuss examples of TFs contributing to the timing of developmental events.

ARF transcription factors regulate lateral root priming in the Quiescent Centre. Temporal auxin oscillations are converted into pulsating gene activation signals by the modulation of ARF activity (Figure 4d) (Bellows et al., 2023). Auxin signaling and thus Aux/IAA degradation dynamics are integrated with shoot‐derived light signals to mark the positions of future lateral roots by discrete gene expression peaks (Kircher & Schopfer, 2018; Reyes‐Hernández & Maizel, 2023). Thus, dynamic signals come together to create discrete temporal outputs.

LAFL TFs play key roles in several developmental transitions, and their ability to induce specific reprogramming events means their expression needs to be temporally restricted (Figure 4d) (Gazzarrini & Song, 2024). LEC1, FUS3, and LEC2 are expressed during the early stages of embryogenesis, where they increase chromatin accessibility to promote embryonic programs (Gazzarrini & Song, 2024; Tao et al., 2017). During embryo maturation, LAFLs then promote seed dormancy, and several LAFL mutants display reduced seed viability (Raz et al., 2001; Wang & Perry, 2013). The LAFLs co‐regulate the timing of the vegetative phase transition, as LEC2 induces embryonic miR156 expression, and FUS3 modulates ethylene response, influencing vegetative growth (Gazzarrini & Song, 2024). Upon vegetative growth, most LAFLs are repressed, and ectopic vegetative expression results in the induction of somatic embryogenesis or production of cotyledon‐like leaves (Gazzarrini et al., 2004; Gazzarrini & Song, 2024; Lotan et al., 1998).

SPL TFs are the master regulators of vegetative phase change, and their protein levels are controlled by miR156 levels (Wu & Poethig, 2006). In turn, SPLs, including SPL9, induce the expression of miR156, thus forming a negative feedback loop (Wu et al., 2009). In juvenile leaves, SPL9 is responsible for inducing a leaf age‐dependent proliferative burst (Li, Jenke, et al., 2024). During the induction of the vegetative phase change, SPL levels are influenced by additional factors such as sugar affecting transcription and ABA signaling, which affects SPL phosphorylation (Dong et al., 2021; Meng et al., 2021). Threshold levels of SPLs then induce the vegetative phase transition in part through the expression of miR172, a miRNA that limits levels of TARGET OF EARLY ACTIVATION TAGGED (EAT) (TOE) 1/2, a TF that represses adult leaf features (Fornara & Coupland, 2009; Poethig & Fouracre, 2024).

LEAFY (LFY) is a pioneer TF that initiates flowering. It can trigger cellular reprogramming and alter gene expression to initiate floral transition even in root explant cells (Jin et al., 2021; Lai et al., 2021). Precocious LFY expression causes premature flowering, and thus, LFY expression timing is carefully regulated through the activities of various flowering factors (Weigel et al., 1992; Weigel & Nilsson, 1995). *LFY* transcription is repressed by the TERMINAL FLOWER 1 (TFL1) – FD complex and promoted when FT competes with TFL1 to prevent binding to and repression of *LFY* (Yamaguchi, 2021; Zhu, Klasfeld, et al., 2020). LFY itself then induces chromatin opening, allowing other TFs to bind and promote floral meristem identity genes (Freytes et al., 2021).

Stomatal differentiation on gynoecial valves is controlled in part by the floral organ regulators AGAMOUS (AG) and SEPALLATA3 (SEP3), with stomatal differentiation timed to coincide with fertilization (Figure 4d) (Brazel et al., 2023). AG and SEP3 suppress *MUTE* transcription, preventing stomatal lineage progression early on in fruit development; over time, decreasing levels of AG and SEP3 allow for *MUTE* transcription and stomatal differentiation upon fertilization (Brazel et al., 2023). AG was previously shown to suppress leaf traits in gynoecial valves, and these findings indicate that stomatal fate progression, but not stomatal fate initiation, is one of those traits (Ó'Maoiléidigh et al., 2018).

The PLT TFs orchestrate the timing of protophloem development in the root meristem by forming a gradient that inhibits PHLOEM EARLY DNA‐BINDING‐WITH‐ONE‐FINGER (PEAR)‐induced phloem differentiation. PLTs prevent early PEAR activity by inhibiting the expression of its target gene ALTERED PHLOEM DEVELOPMENT (APL) (Roszak et al., 2021). As PLT levels decrease, PEAR TFs can induce the expression of APL and promote differentiation; in this process, PLT2 is necessary and sufficient, as ectopic PLT2 expression can delay phloem differentiation (Roszak et al., 2021).

While the TFs mentioned above are pivotal, they do not act in isolation and instead are part of broader, interconnected gene regulatory networks that act together with other signaling pathways and TFs. This ensures precise spatiotemporal coordination of plant development.

## CONCLUSION AND PERSPECTIVES

Plant body plans develop robustly under the control of a myriad of developmental regulators. Thus far, the timing of plant development has mainly been studied in the context of phase transitions, but recent insights indicate that temporal regulation also exists on a local scale and controls or limits cell identity and patterning in specific situations. One of the challenges in identifying temporal control on the local scale is that this control seems often not to be present in all instances or contexts: a trend in the current examples is that temporal control is limited to specific tissue types or developmental stages. We anticipate that studying cell identity across more contexts will reveal additional examples of temporal regulation.

Temporal regulation of plant development can further be unraveled using new technical advancements. Single cell or single nucleus omics technologies enable a more detailed look into cell identity and allow better tracking of identity changes (Adema et al., 2024; Nolan & Shahan, 2023; Ryu et al., 2021; Swift et al., 2022). These approaches can also help elucidate complex cell identities such as mixed identities or cell identity variation between tissues (Kim et al., 2021; Lee et al., 2023; Petersen et al., 2024; Shahan et al., 2022). Answering questions on the temporal regulation of phase or identity transitions requires studying these changes in their natural context as much as possible. Spatial omics and multiplexed FISH approaches now allow for visualization of many transcripts, while advances in time course and time lapse imaging enable tracking cells in their native context over time (Adema et al., 2024; Harline & Roeder, 2023; Nobori et al., 2023; Nolan & Shahan, 2023). Finally, novel CRISPR technologies allow for lineage tracing and spatiotemporally specific knockout of fate regulators (Decaestecker et al., 2019; Donà et al., 2023; Gehrke et al., 2023). Now that we are touching upon the temporal restriction and regulation of cell identity more and more, these technologies can help better define, identify, and track cell identities as well as manipulate them in a more targeted way to separate temporal from other, spatial, effects.

In conclusion, the timing of developmental events has been studied at both systemic and local scales. Systemic changes, phase transitions, that affect the whole plant have been studied in some detail and more mechanisms are being unraveled. By contrast, local regulation, where timing is controlled at a cell or tissue level, is now being investigated more and more. It will be exciting to see when, where, and how local developmental timing is being regulated (Boxes 1 and 2).

Box 1 **Bullet point summary**

- Temporal regulation in plants has been examined in the systemic context of phase transitions, and their regulators have been studied extensively.
- Control of developmental timing at a local scale, limiting local organ or cell identity transitions, has gained attention in recent years, but separating temporal from spatial control remains challenging.
- Environmental signals and tissue context play large roles in controlling the developmental timing of plant phase transitions and cell identity regulation.
- Molecular mechanisms controlling the timing of developmental events are varied and complex, and often act through the integration of multiple factors.

Box 2 **Open questions**

1. How are the effects of the multiple factors that control each plant phase transition weighted and integrated?
2. At what stages and in which cell types and organs are cell identity and patterning temporally regulated during plant development?
3. How common is temporal regulation independently from spatial, tissue, and environmental factors?
4. What molecular mechanisms are employed in the temporal regulation of cell identity? How do they determine the correct timing of developmental events?
5. Are there common mechanisms that control the local timing of development across tissues or developmental stages?

## CONFLICT OF INTEREST

The authors declare no conflicts of interest.

## Data Availability

Data sharing is not applicable to this article as no new data were created or analyzed in this study.

## References

[tpj70130-bib-0001] Abarca, A. , Franck, C.M. & Zipfel, C. (2021) Family‐wide evaluation of RAPID ALKALINIZATION FACTOR peptides. Plant Physiology, 187, 996–1010. Available from: 10.1093/plphys/kiab308 34608971 PMC8491022

[tpj70130-bib-0002] Adema, K. , Schon, M.A. , Nodine, M.D. & Kohlen, W. (2024) Lost in space: what single‐cell RNA sequencing cannot tell you. Trends in Plant Science, 29, 1018–1028. Available from: 10.1016/j.tplants.2024.03.010 38570278

[tpj70130-bib-0003] Aida, M. , Beis, D. , Heidstra, R. , Willemsen, V. , Blilou, I. , Galinha, C. et al. (2004) The PLETHORA genes mediate patterning of the Arabidopsis root stem cell niche. Cell, 119, 109–120. Available from: 10.1016/j.cell.2004.09.018 15454085

[tpj70130-bib-0004] Aliaga Fandino, A.C. & Hardtke, C.S. (2022) Auxin transport in developing protophloem: a case study in canalization. Journal of Plant Physiology, 269, 153594. Available from: 10.1016/j.jplph.2021.153594 34953411

[tpj70130-bib-0005] Aliaga Fandino, A.C. , Jelínková, A. , Marhava, P. , Petrášek, J. & Hardtke, C.S. (2024) Ectopic assembly of an auxin efflux control machinery shifts developmental trajectories. Plant Cell, 36, 1791–1805. Available from: 10.1093/plcell/koae023 38267818 PMC11062438

[tpj70130-bib-0006] Alvarez, J.M. , Brooks, M.D. , Swift, J. & Coruzzi, G.M. (2021) Time‐based systems biology approaches to capture and model dynamic gene regulatory networks. Annual Review of Plant Biology, 72, 105–131. Available from: 10.1146/annurev-arplant-081320-090914 PMC9312366 33667112

[tpj70130-bib-0007] Ay, N. , Irmler, K. , Fischer, A. , Uhlemann, R. , Reuter, G. & Humbeck, K. (2009) Epigenetic programming via histone methylation at WRKY53 controls leaf senescence in *Arabidopsis thaliana* . The Plant Journal: For Cell and Molecular Biology, 58, 333–346. Available from: 10.1111/j.1365-313X.2008.03782.x 19143996

[tpj70130-bib-0008] Balasubramanian, S. , Sureshkumar, S. , Lempe, J. & Weigel, D. (2006) Potent induction of *Arabidopsis thaliana* flowering by elevated growth temperature. PLoS Genetics, 2, e106. Available from: 10.1371/journal.pgen.0020106 16839183 PMC1487179

[tpj70130-bib-0009] Bastow, R. , Mylne, J.S. , Lister, C. , Lippman, Z. , Martienssen, R.A. & Dean, C. (2004) Vernalization requires epigenetic silencing of FLC by histone methylation. Nature, 427, 164–167. Available from: 10.1038/nature02269 14712277

[tpj70130-bib-0010] Bellows, S. , Janes, G. , Avitabile, D. , King, J.R. , Bishopp, A. & Farcot, E. (2023) Fluctuations in auxin levels depend upon synchronicity of cell divisions in a one‐dimensional model of auxin transport. PLoS Computational Biology, 19, e1011646. Available from: 10.1371/journal.pcbi.1011646 38032890 PMC10688697

[tpj70130-bib-0011] Bente, H. & Köhler, C. (2024) Molecular basis and evolutionary drivers of endosperm‐based hybridization barriers. Plant Physiology, 195, 155–169. Available from: 10.1093/plphys/kiae050 38298124 PMC11060687

[tpj70130-bib-0012] Bentsink, L. & Koornneef, M. (2008) Seed dormancy and germination. Arabidopsis Book, 6, e0119. Available from: 10.1199/tab.0119 22303244 PMC3243337

[tpj70130-bib-0013] Berckmans, B. , Kirschner, G. , Gerlitz, N. , Stadler, R. & Simon, R. (2020) CLE40 signaling regulates root stem cell fate. Plant Physiology, 182, 1776–1792. Available from: 10.1104/pp.19.00914 31806736 PMC7140941

[tpj70130-bib-0014] Bergmann, D.C. & Sack, F.D. (2007) Stomatal development. Annual Review of Plant Biology, 58, 163–181. Available from: 10.1146/annurev.arplant.58.032806.104023 17201685

[tpj70130-bib-0015] Birnbaum, K.D. & Roudier, F. (2017) Epigenetic memory and cell fate reprogramming in plants. Regeneration (Oxford, England), 4, 15–20. Available from: 10.1002/reg2.73 28316791 PMC5350078

[tpj70130-bib-0016] Birnie, A. , Plat, A. , Korkmaz, C. & Bothma, J.P. (2023) Precisely timed regulation of enhancer activity defines the binary expression pattern of Fushi tarazu in the *Drosophila* embryo. Current Biology: CB, 33, 2839–2850. Available from: 10.1016/j.cub.2023.04.005 37116484 PMC10373528

[tpj70130-bib-0017] Blázquez, M.A. , Soowal, L.N. , Lee, I. & Weigel, D. (1997) LEAFY expression and flower initiation in Arabidopsis. Development, 124, 3835–3844. Available from: 10.1242/dev.124.19.3835 9367439

[tpj70130-bib-0018] Bouyer, D. , Kramdi, A. , Kassam, M. , Heese, M. , Schnittger, A. , Roudier, F. et al. (2017) DNA methylation dynamics during early plant life. Genome Biology, 18, 179. Available from: 10.1186/s13059-017-1313-0 28942733 PMC5611644

[tpj70130-bib-0019] Brazel, A.J. , Fattorini, R. , McCarthy, J. , Franzen, R. , Rümpler, F. , Coupland, G. et al. (2023) AGAMOUS mediates timing of guard cell formation during gynoecium development. PLoS Genetics, 19, e1011000. Available from: 10.1371/journal.pgen.1011000 37819989 PMC10593234

[tpj70130-bib-0020] Buijs, G. (2020) A perspective on secondary seed dormancy in *Arabidopsis thaliana* . Plants (Basel, Switzerland), 9, 749. Available from: 10.3390/plants9060749 32549219 PMC7355504

[tpj70130-bib-0021] Butel, N. , Qiu, Y. , Xu, W. , Santos‐González, J. & Köhler, C. (2024) Parental conflict driven regulation of endosperm cellularization by a family of auxin response factors. Nature Plants, 10, 1018–1026. Available from: 10.1038/s41477-024-01706-y 38806655 PMC11208147

[tpj70130-bib-0022] Carbonnel, S. , Cornelis, S. & Hazak, O. (2023) The CLE33 peptide represses phloem differentiation via autocrine and paracrine signaling in Arabidopsis. Communications Biology, 6, 588. Available from: 10.1038/s42003-023-04972-2 37280369 PMC10244433

[tpj70130-bib-0023] Cerbantez‐Bueno, V.E. , Serwatowska, J. , Rodríguez‐Ramos, C. , Cruz‐Valderrama, J.E. & de Folter, S. (2024) The role of D3‐type cyclins is related to cytokinin and the bHLH transcription factor SPATULA in Arabidopsis gynoecium development. Planta, 260, 48. Available from: 10.1007/s00425-024-04481-4 38980389 PMC11233295

[tpj70130-bib-0024] Chen, Y. , Ince, Y.Ç. , Kawamura, A. , Favero, D.S. , Suzuki, T. & Sugimoto, K. (2024) ELONGATED HYPOCOTYL5‐mediated light signaling promotes shoot regeneration in *Arabidopsis thaliana* . Plant Physiology, 196(4), kiae474. Available from: 10.1093/plphys/kiae474 39315875

[tpj70130-bib-0025] Cho, L.‐H. , Yoon, J. & An, G. (2017) The control of flowering time by environmental factors. The Plant Journal, 90, 708–719. Available from: 10.1111/tpj.13461 27995671

[tpj70130-bib-0026] Clark, E. , Battistara, M. & Benton, M.A. (2022) A timer gene network is spatially regulated by the terminal system in the *Drosophila* embryo. eLife, 11, e78902. Available from: 10.7554/eLife.78902 36524728 PMC10065802

[tpj70130-bib-0027] Clark, E. , Peel, A.D. & Akam, M. (2019) Arthropod segmentation. Development (Cambridge, England), 146, dev170480. Available from: 10.1242/dev.170480 31554626

[tpj70130-bib-0028] Coen, E. & Prusinkiewicz, P. (2024) Developmental timing in plants. Nature Communications, 15, 2674. Available from: 10.1038/s41467-024-46941-1 PMC10965974 38531864

[tpj70130-bib-0029] Corbesier, L. , Vincent, C. , Jang, S. , Fornara, F. , Fan, Q. , Searle, I. et al. (2007) FT protein movement contributes to long‐distance signaling in floral induction of Arabidopsis. Science, 316, 1030–1033. Available from: 10.1126/science.1141752 17446353

[tpj70130-bib-0030] Csorba, T. , Questa, J.I. , Sun, Q. & Dean, C. (2014) Antisense COOLAIR mediates the coordinated switching of chromatin states at FLC during vernalization. Proceedings of the National Academy of Sciences of the United States of America, 111, 16160–16165. Available from: 10.1073/pnas.1419030111 25349421 PMC4234544

[tpj70130-bib-0031] D'Ario, M. , Griffiths‐Jones, S. & Kim, M. (2017) Small RNAs: big impact on plant development. Trends in Plant Science, 22, 1056–1068. Available from: 10.1016/j.tplants.2017.09.009 29032035

[tpj70130-bib-0032] D'Ario, M. , Tavares, R. , Schiessl, K. , Desvoyes, B. , Gutierrez, C. , Howard, M. et al. (2021) Cell size controlled in plants using DNA content as an internal scale. Science, 372, 1176–1181. Available from: 10.1126/science.abb4348 34112688

[tpj70130-bib-0033] Decaestecker, W. , Buono, R.A. , Pfeiffer, M.L. , Vangheluwe, N. , Jourquin, J. , Karimi, M. et al. (2019) CRISPR‐TSKO: a technique for efficient mutagenesis in specific cell types, tissues, or organs in Arabidopsis. Plant Cell, 31, 2868–2887. Available from: 10.1105/tpc.19.00454 31562216 PMC6925012

[tpj70130-bib-0034] Denay, G. , Creff, A. , Moussu, S. , Wagnon, P. , Thévenin, J. , Gérentes, M.‐F. et al. (2014) Endosperm breakdown in Arabidopsis requires heterodimers of the basic helix‐loop‐helix proteins ZHOUPI and INDUCER OF CBP EXPRESSION 1. Development (Cambridge, England), 141, 1222–1227. Available from: 10.1242/dev.103531 24553285

[tpj70130-bib-0035] Diaz‐Cuadros, M. , Miettinen, T.P. , Skinner, O.S. , Sheedy, D. , Díaz‐García, C.M. , Gapon, S. et al. (2023) Metabolic regulation of species‐specific developmental rates. Nature, 613, 550–557. Available from: 10.1038/s41586-022-05574-4 36599986 PMC9944513

[tpj70130-bib-0036] Doe, C.Q. (2017) Temporal patterning in the *Drosophila* CNS. Annual Review of Cell and Developmental Biology, 33, 219–240. Available from: 10.1146/annurev-cellbio-111315-125210 28992439

[tpj70130-bib-0037] Doll, N.M. & Ingram, G.C. (2022) Embryo‐endosperm interactions. Annual Review of Plant Biology, 73, 293–321. Available from: 10.1146/annurev-arplant-102820-091838 35130443

[tpj70130-bib-0038] Doll, N.M. , Van Hautegem, T. , Schilling, N. , De Rycke, R. , Winter, F. , Fendrych, M. et al. (2023) Endosperm cell death promoted by NAC transcription factors facilitates embryo invasion in Arabidopsis. Current Biology: CB, 33, 3785–3795. Available from: 10.1016/j.cub.2023.08.003 37633282 PMC7615161

[tpj70130-bib-0039] Donà, M. , Bradamante, G. , Bogojevic, Z. , Gutzat, R. , Streubel, S. , Mosiolek, M. et al. (2023) A versatile CRISPR‐based system for lineage tracing in living plants. The Plant Journal: For Cell and Molecular Biology, 115, 1169–1184. Available from: 10.1111/tpj.16378 37403571

[tpj70130-bib-0040] Dong, H. , Yan, S. , Jing, Y. , Yang, R. , Zhang, Y. , Zhou, Y. et al. (2021) MIR156‐targeted SPL9 is phosphorylated by SnRK2s and interacts with ABI5 to enhance ABA responses in Arabidopsis. Frontiers in Plant Science, 12, 708573. Available from: 10.3389/fpls.2021.708573 34367226 PMC8334859

[tpj70130-bib-0041] Doody, E. , Zha, Y. , He, J. & Poethig, R.S. (2022) The genetic basis of natural variation in the timing of vegetative phase change in *Arabidopsis thaliana* . Development (Cambridge, England), 149, dev200321. Available from: 10.1242/dev.200321 35502761 PMC9233309

[tpj70130-bib-0042] Dorrity, M.W. , Alexandre, C.M. , Hamm, M.O. , Vigil, A.‐L. , Fields, S. , Queitsch, C. et al. (2021) The regulatory landscape of *Arabidopsis thaliana* roots at single‐cell resolution. Nature Communications, 12, 3334. Available from: 10.1038/s41467-021-23675-y PMC8184767 34099698

[tpj70130-bib-0043] Durgaprasad, K. , Roy, M.V. , Venugopal, M.A. , Kareem, A. , Raj, K. , Willemsen, V. et al. (2019) Gradient expression of transcription factor imposes a boundary on organ regeneration potential in plants. Cell Reports, 29, 453–463.e3. Available from: 10.1016/j.celrep.2019.08.099 31597103

[tpj70130-bib-0044] El‐Danaf, R.N. , Rajesh, R. & Desplan, C. (2023) Temporal regulation of neural diversity in *Drosophila* and vertebrates. Seminars in Cell & Developmental Biology, 142, 13–22. Available from: 10.1016/j.semcdb.2022.05.011 35623984 PMC11585012

[tpj70130-bib-0045] Etchells, J.P. & Turner, S.R. (2010) The PXY‐CLE41 receptor ligand pair defines a multifunctional pathway that controls the rate and orientation of vascular cell division. Development (Cambridge, England), 137, 767–774. Available from: 10.1242/dev.044941 20147378

[tpj70130-bib-0046] Ezaki, K. , Koga, H. , Takeda‐Kamiya, N. , Toyooka, K. , Higaki, T. , Sakamoto, S. et al. (2024) Precocious cell differentiation occurs in proliferating cells in leaf primordia in Arabidopsis angustifolia3 mutant. Frontiers in Plant Science, 15, 1322223. Available from: 10.3389/fpls.2024.1322223 38689848 PMC11058843

[tpj70130-bib-0047] Finkelstein, R. , Reeves, W. , Ariizumi, T. & Steber, C. (2008) Molecular aspects of seed dormancy. Annual Review of Plant Biology, 59, 387–415. Available from: 10.1146/annurev.arplant.59.032607.092740 18257711

[tpj70130-bib-0048] Fornara, F. & Coupland, G. (2009) Plant phase transitions make a SPLash. Cell, 138, 625–627. Available from: 10.1016/j.cell.2009.08.011 19703391

[tpj70130-bib-0049] Freytes, S.N. , Canelo, M. & Cerdán, P.D. (2021) Regulation of flowering time: when and where? Current Opinion in Plant Biology, 63, 102049. Available from: 10.1016/j.pbi.2021.102049 33975153

[tpj70130-bib-0050] Galinha, C. , Hofhuis, H. , Luijten, M. , Willemsen, V. , Blilou, I. , Heidstra, R. et al. (2007) PLETHORA proteins as dose‐dependent master regulators of Arabidopsis root development. Nature, 449, 1053–1057. Available from: 10.1038/nature06206 17960244

[tpj70130-bib-0051] Gao, J. , Zhang, K. , Cheng, Y.‐J. , Yu, S. , Shang, G.‐D. , Wang, F.‐X. et al. (2022) A robust mechanism for resetting juvenility during each generation in Arabidopsis. Nature Plants, 8, 257–268. Available from: 10.1038/s41477-022-01110-4 35318444

[tpj70130-bib-0052] Gazzarrini, S. & Song, L. (2024) LAFL factors in seed development and phase transitions. Annual Review of Plant Biology, 75, 459–488. Available from: 10.1146/annurev-arplant-070623-111458 38657282

[tpj70130-bib-0053] Gazzarrini, S. , Tsuchiya, Y. , Lumba, S. , Okamoto, M. & McCourt, P. (2004) The transcription factor FUSCA3 controls developmental timing in Arabidopsis through the hormones gibberellin and abscisic acid. Developmental Cell, 7, 373–385. Available from: 10.1016/j.devcel.2004.06.017 15363412

[tpj70130-bib-0054] Gehrke, F. , Ruiz‐Duarte, P. , Schindele, A. , Wolf, S. & Puchta, H. (2023) An inducible CRISPR‐kill system for temporally controlled cell type‐specific cell ablation in *Arabidopsis thaliana* . The New Phytologist, 239, 2041–2052. Available from: 10.1111/nph.19102 37381079

[tpj70130-bib-0055] Gerber, T. , Murawala, P. , Knapp, D. , Masselink, W. , Schuez, M. , Hermann, S. et al. (2018) Single‐cell analysis uncovers convergence of cell identities during axolotl limb regeneration. Science, 362, eaaq0681. Available from: 10.1126/science.aaq0681 30262634 PMC6669047

[tpj70130-bib-0056] Gomez, C. , Ozbudak, E.M. , Wunderlich, J. , Baumann, D. , Lewis, J. & Pourquié, O. (2008) Control of segment number in vertebrate embryos. Nature, 454, 335–339. Available from: 10.1038/nature07020 18563087

[tpj70130-bib-0057] Gong, Y. , Dale, R. , Fung, H.F. , Amador, G.O. , Smit, M.E. & Bergmann, D.C. (2023) A cell size threshold triggers commitment to stomatal fate in Arabidopsis. Science Advances, 9, eadf3497. Available from: 10.1126/sciadv.adf3497 37729402 PMC10881030

[tpj70130-bib-0058] González‐Suárez, P. , Walker, C.H. & Bennett, T. (2020) Bloom and bust: understanding the nature and regulation of the end of flowering. Current Opinion in Plant Biology, 57, 24–30. Available from: 10.1016/j.pbi.2020.05.009 32619967

[tpj70130-bib-0059] González‐Suárez, P. , Walker, C.H. , Lock, T. & Bennett, T. (2024) FLOWERING LOCUS T‐mediated thermal signalling regulates age‐dependent inflorescence development in *Arabidopsis thaliana* . Journal of Experimental Botany, 75, 4400–4414. Available from: 10.1093/jxb/erae094 38442244 PMC11263484

[tpj70130-bib-0060] Grbić, V. & Bleecker, A.B. (1995) Ethylene regulates the timing of leaf senescence in Arabidopsis. The Plant Journal, 8, 595–602. Available from: 10.1046/j.1365-313X.1995.8040595.x

[tpj70130-bib-0061] Grosskortenhaus, R. , Pearson, B.J. , Marusich, A. & Doe, C.Q. (2005) Regulation of temporal identity transitions in *Drosophila* neuroblasts. Developmental Cell, 8, 193–202. Available from: 10.1016/j.devcel.2004.11.019 15691761

[tpj70130-bib-0062] Hamant, O. , Heisler, M.G. , Jönsson, H. , Krupinski, P. , Uyttewaal, M. , Bokov, P. et al. (2008) Developmental patterning by mechanical signals in Arabidopsis. Science, 322, 1650–1655. Available from: 10.1126/science.1165594 19074340

[tpj70130-bib-0063] Han, H. , Liu, X. & Zhou, Y. (2020) Transcriptional circuits in control of shoot stem cell homeostasis. Current Opinion in Plant Biology, 53, 50–56. Available from: 10.1016/j.pbi.2019.10.004 31766002

[tpj70130-bib-0064] Han, S.‐K. , Herrmann, A. , Yang, J. , Iwasaki, R. , Sakamoto, T. , Desvoyes, B. et al. (2022) Deceleration of the cell cycle underpins a switch from proliferative to terminal divisions in plant stomatal lineage. Developmental Cell, 57, 569–582.e6. Available from: 10.1016/j.devcel.2022.01.014 35148836 PMC8926846

[tpj70130-bib-0065] Harima, Y. , Takashima, Y. , Ueda, Y. , Ohtsuka, T. & Kageyama, R. (2013) Accelerating the tempo of the segmentation clock by reducing the number of introns in the Hes7 gene. Cell Reports, 3, 1–7. Available from: 10.1016/j.celrep.2012.11.012 23219549

[tpj70130-bib-0066] Harline, K. & Roeder, A.H.K. (2023) An optimized pipeline for live imaging whole Arabidopsis leaves at cellular resolution. Plant Methods, 19, 10. Available from: 10.1186/s13007-023-00987-2 36726130 PMC9890716

[tpj70130-bib-0067] He, J. , Xu, M. , Willmann, M.R. , McCormick, K. , Hu, T. , Yang, L. et al. (2018) Threshold‐dependent repression of SPL gene expression by miR156/miR157 controls vegetative phase change in *Arabidopsis thaliana* . PLoS Genetics, 14, e1007337. Available from: 10.1371/journal.pgen.1007337 29672610 PMC5929574

[tpj70130-bib-0068] He, L. , Huang, H. , Bradai, M. , Zhao, C. , You, Y. , Ma, J. et al. (2022) DNA methylation‐free Arabidopsis reveals crucial roles of DNA methylation in regulating gene expression and development. Nature Communications, 13, 1335. Available from: 10.1038/s41467-022-28940-2 PMC8921224 35288562

[tpj70130-bib-0069] Hehenberger, E. , Kradolfer, D. & Köhler, C. (2012) Endosperm cellularization defines an important developmental transition for embryo development. Development (Cambridge, England), 139, 2031–2039. Available from: 10.1242/dev.077057 22535409

[tpj70130-bib-0070] Hemenway, E.A. & Gehring, M. (2023) Epigenetic regulation during plant development and the capacity for epigenetic memory. Annual Review of Plant Biology, 74, 87–109. Available from: 10.1146/annurev-arplant-070122-025047 PMC10280588 36854474

[tpj70130-bib-0071] Homem, C.C.F. & Knoblich, J.A. (2012) *Drosophila* neuroblasts: a model for stem cell biology. Development (Cambridge, England), 139, 4297–4310. Available from: 10.1242/dev.080515 23132240

[tpj70130-bib-0072] Horiguchi, G. , Van Lijsebettens, M. , Candela, H. , Micol, J.L. & Tsukaya, H. (2012) Ribosomes and translation in plant developmental control. Plant Science: An International Journal of Experimental Plant Biology, 191, 24–34. Available from: 10.1016/j.plantsci.2012.04.008 22682562

[tpj70130-bib-0073] Huang, R. , Huang, T. & Irish, V.F. (2021) Do epigenetic timers control petal development? Frontiers in Plant Science, 12, 709360. Available from: 10.3389/fpls.2021.709360 34295349 PMC8290480

[tpj70130-bib-0074] Huang, X. & Sun, M.‐X. (2022) H3K27 methylation regulates the fate of two cell lineages in male gametophytes. Plant Cell, 34, 2989–3005. Available from: 10.1093/plcell/koac136 35543471 PMC9338816

[tpj70130-bib-0075] Ikeuchi, M. , Favero, D.S. , Sakamoto, Y. , Iwase, A. , Coleman, D. , Rymen, B. et al. (2019) Molecular mechanisms of plant regeneration. Annual Review of Plant Biology, 70, 377–406. Available from: 10.1146/annurev-arplant-050718-100434 30786238

[tpj70130-bib-0076] Ikeuchi, M. , Iwase, A. , Rymen, B. , Harashima, H. , Shibata, M. , Ohnuma, M. et al. (2015) PRC2 represses dedifferentiation of mature somatic cells in Arabidopsis. Nature Plants, 1, 15089. Available from: 10.1038/nplants.2015.89 27250255

[tpj70130-bib-0077] Ikeuchi, M. , Iwase, A. & Sugimoto, K. (2015) Control of plant cell differentiation by histone modification and DNA methylation. Current Opinion in Plant Biology, 28, 60–67. Available from: 10.1016/j.pbi.2015.09.004 26454697

[tpj70130-bib-0078] Iwata, R. , Casimir, P. , Erkol, E. , Boubakar, L. , Planque, M. , Gallego López, I.M. et al. (2023) Mitochondria metabolism sets the species‐specific tempo of neuronal development. Science, 379, eabn4705. Available from: 10.1126/science.abn4705 36705539

[tpj70130-bib-0079] Jin, R. , Klasfeld, S. , Zhu, Y. , Fernandez Garcia, M. , Xiao, J. , Han, S.‐K. et al. (2021) LEAFY is a pioneer transcription factor and licenses cell reprogramming to floral fate. Nature Communications, 12, 626. Available from: 10.1038/s41467-020-20883-w PMC7840934 33504790

[tpj70130-bib-0080] Jin, S. & Ahn, J.H. (2021) Regulation of flowering time by ambient temperature: repressing the repressors and activating the activators. The New Phytologist, 230, 938–942. Available from: 10.1111/nph.17217 33474759

[tpj70130-bib-0081] Johnson, M.H. & Day, M.L. (2000) Egg timers: how is developmental time measured in the early vertebrate embryo? BioEssays news rev. Molecular, Cellular, and Developmental Biology, 22, 57–63. Available from: 10.1002/(SICI)1521-1878(200001)22:1<57::AID-BIES10>3.0.CO;2-L 10649291

[tpj70130-bib-0082] Jones, A.R. , Band, L.R. & Murray, J.A.H. (2019) Double or nothing? Cell division and cell size control. Trends in Plant Science, 24, 1083–1093. Available from: 10.1016/j.tplants.2019.09.005 31630972

[tpj70130-bib-0083] Kareem, A. , Radhakrishnan, D. , Sondhi, Y. , Aiyaz, M. , Roy, M.V. , Sugimoto, K. et al. (2016) De novo assembly of plant body plan: a step ahead of deadpool. Regeneration, 3, 182–197. Available from: 10.1002/reg2.68 27800169 PMC5084358

[tpj70130-bib-0084] Kawakatsu, T. , Nery, J.R. , Castanon, R. & Ecker, J.R. (2017) Dynamic DNA methylation reconfiguration during seed development and germination. Genome Biology, 18, 171. Available from: 10.1186/s13059-017-1251-x 28911331 PMC5599895

[tpj70130-bib-0085] Kim, C.M. , Han, C. & Dolan, L. (2017) RSL class I genes positively regulate root hair development in *Oryza sativa* . The New Phytologist, 213, 314–323. Available from: 10.1111/nph.14160 27716929

[tpj70130-bib-0086] Kim, E.‐D. , Dorrity, M.W. , Fitzgerald, B.A. , Seo, H. , Sepuru, K.M. , Queitsch, C. et al. (2022) Dynamic chromatin accessibility deploys heterotypic cis/trans‐acting factors driving stomatal cell‐fate commitment. Nature Plants, 8, 1453–1466. Available from: 10.1038/s41477-022-01304-w 36522450 PMC9788986

[tpj70130-bib-0087] Kim, H.J. , Park, J.‐H. , Kim, J. , Kim, J.J. , Hong, S. , Kim, J. et al. (2018) Time‐evolving genetic networks reveal a NAC troika that negatively regulates leaf senescence in Arabidopsis. Proceedings of the National Academy of Sciences of the United States of America, 115, E4930–E4939. Available from: 10.1073/pnas.1721523115 29735710 PMC6003463

[tpj70130-bib-0088] Kim, J.‐Y. , Symeonidi, E. , Pang, T.Y. , Denyer, T. , Weidauer, D. , Bezrutczyk, M. et al. (2021) Distinct identities of leaf phloem cells revealed by single cell transcriptomics. Plant Cell, 33, 511–530. Available from: 10.1093/plcell/koaa060 33955487 PMC8136902

[tpj70130-bib-0089] Kim, J. , Kim, J.H. , Lyu, J.I. , Woo, H.R. & Lim, P.O. (2018) New insights into the regulation of leaf senescence in Arabidopsis. Journal of Experimental Botany, 69, 787–799. Available from: 10.1093/jxb/erx287 28992051

[tpj70130-bib-0090] Kim, J.‐Y. , Yang, W. , Forner, J. , Lohmann, J.U. , Noh, B. & Noh, Y. (2018) Epigenetic reprogramming by histone acetyltransferase HAG1/AtGCN5 is required for pluripotency acquisition in Arabidopsis. The EMBO Journal, 37, e98726. Available from: 10.15252/embj.201798726 30061313 PMC6187204

[tpj70130-bib-0091] Kim, S.Y. , Zhu, T. & Sung, Z.R. (2010) Epigenetic regulation of gene programs by EMF1 and EMF2 in Arabidopsis. Plant Physiology, 152, 516–528. Available from: 10.1104/pp.109.143495 19783648 PMC2815887

[tpj70130-bib-0092] Kircher, S. & Schopfer, P. (2018) The plant hormone auxin beats the time for oscillating light‐regulated lateral root induction. Development, 145, dev169839. Available from: 10.1242/dev.169839 30389851

[tpj70130-bib-0093] Kleiber, M. (1947) Body size and metabolic rate. Physiological Reviews, 27, 511–541. Available from: 10.1152/physrev.1947.27.4.511 20267758

[tpj70130-bib-0094] Kong, S. , Zhu, M. , Pan, D. , Lane, B. , Smith, R.S. & Roeder, A.H.K. (2024) Tradeoff between speed and robustness in primordium initiation mediated by auxin‐CUC1 interaction. *bioRxiv*. 2023.11.30.569401. Available from: 10.1101/2023.11.30.569401 PMC11246466 39003301

[tpj70130-bib-0095] Kong, S. , Zhu, M. , Scarpin, M.R. , Pan, D. , Jia, L. , Martinez, R.E. et al. (2024) DRMY1 promotes robust morphogenesis in Arabidopsis by sustaining the translation of cytokinin‐signaling inhibitor proteins. Developmental Cell, 59(23), S1534580724005124. Available from: 10.1016/j.devcel.2024.08.010 PMC11614703 39305905

[tpj70130-bib-0096] Lai, X. , Blanc‐Mathieu, R. , GrandVuillemin, L. , Huang, Y. , Stigliani, A. , Lucas, J. et al. (2021) The LEAFY floral regulator displays pioneer transcription factor properties. Molecular Plant, 14, 829–837. Available from: 10.1016/j.molp.2021.03.004 33684542

[tpj70130-bib-0097] Larriba, E. , Sánchez‐García, A.B. , Martínez‐Andújar, C. , Albacete, A. & Pérez‐Pérez, J.M. (2021) Tissue‐specific metabolic reprogramming during wound‐induced organ formation in tomato hypocotyl explants. International Journal of Molecular Sciences, 22, 10112. Available from: 10.3390/ijms221810112 34576275 PMC8466849

[tpj70130-bib-0098] Lee, J. , Kang, M.H. , Kim, J.Y. & Lim, P.O. (2021) The role of light and circadian clock in regulation of leaf senescence. Frontiers in Plant Science, 12, 669170. Available from: 10.3389/fpls.2021.669170 33912212 PMC8075161

[tpj70130-bib-0099] Lee, J.E. , Goretti, D. , Neumann, M. , Schmid, M. & You, Y. (2020) A gibberellin methyltransferase modulates the timing of floral transition at the Arabidopsis shoot meristem. Physiologia Plantarum, 170, 474–487. Available from: 10.1111/ppl.13146 32483836

[tpj70130-bib-0100] Lee, J.‐H. , Jung, J.‐H. & Park, C.‐M. (2015) INDUCER OF CBF EXPRESSION 1 integrates cold signals into FLOWERING LOCUS C‐mediated flowering pathways in Arabidopsis. The Plant Journal: For Cell and Molecular Biology, 84, 29–40. Available from: 10.1111/tpj.12956 26248809

[tpj70130-bib-0101] Lee, L.R. , Guillotin, B. , Rahni, R. , Hutchison, C. , Desvoyes, B. , Gutierrez, C. et al. (2025) Glutathione accelerates the cell cycle and cellular reprogramming in plant regeneration. Developmental Cell. Available from: 10.1016/j.devcel.2024.12.019 ahead of print. PMC12278113 39755116

[tpj70130-bib-0102] Lee, S. , Park, Y.S. , Rhee, J.H. , Chu, H. , Frost, J.M. & Choi, Y. (2024) Insights into plant regeneration: cellular pathways and DNA methylation dynamics. Plant Cell Reports, 43, 120. Available from: 10.1007/s00299-024-03216-9 38634973 PMC11026228

[tpj70130-bib-0103] Lee, T.A. , Nobori, T. , Illouz‐Eliaz, N. , Xu, J. , Jow, B. , Nery, J.R. et al. (2023) A single‐nucleus atlas of seed‐to‐seed development in Arabidopsis. *bioRxiv*. Available from: 10.1101/2023.03.23.533992

[tpj70130-bib-0104] Li, J. , Xu, X. , Tiwari, M. , Chen, Y. , Fuller, M. , Bansal, V. et al. (2021) SPT6 promotes epidermal differentiation and blockade of an intestinal‐like phenotype through control of transcriptional elongation. Nature Communications, 12, 784. Available from: 10.1038/s41467-021-21067-w PMC7862286 33542242

[tpj70130-bib-0105] Li, J. , Zhang, Q. , Wang, Z. & Liu, Q. (2024) The roles of epigenetic regulators in plant regeneration: exploring patterns amidst complex conditions. Plant Physiology, 194, 2022–2038. Available from: 10.1093/plphys/kiae042 38290051 PMC10980418

[tpj70130-bib-0106] Li, W. , Liu, H. , Cheng, Z.J. , Su, Y.H. , Han, H.N. , Zhang, Y. et al. (2011) DNA methylation and histone modifications regulate de novo shoot regeneration in Arabidopsis by modulating WUSCHEL expression and auxin signaling. PLoS Genetics, 7, e1002243. Available from: 10.1371/journal.pgen.1002243 21876682 PMC3158056

[tpj70130-bib-0107] Li, X. , Liang, T. & Liu, H. (2022) How plants coordinate their development in response to light and temperature signals. Plant Cell, 34, 955–966. Available from: 10.1093/plcell/koab302 34904672 PMC8894937

[tpj70130-bib-0108] Li, X.‐M. , Jenke, H. , Strauss, S. , Bazakos, C. , Mosca, G. , Lymbouridou, R. et al. (2024) Cell‐cycle‐linked growth reprogramming encodes developmental time into leaf morphogenesis. Current Biology: CB, 34, 541–556. Available from: 10.1016/j.cub.2023.12.050 38244542

[tpj70130-bib-0109] Liew, L.C. , You, Y. , Auroux, L. , Oliva, M. , Peirats‐Llobet, M. , Ng, S. et al. (2024) Establishment of single‐cell transcriptional states during seed germination. Nature Plants, 10, 1418–1434. Available from: 10.1038/s41477-024-01771-3 39256563 PMC11410669

[tpj70130-bib-0110] Lim, B. , Fukaya, T. , Heist, T. & Levine, M. (2018) Temporal dynamics of pair‐rule stripes in living *Drosophila* embryos. Proceedings of the National Academy of Sciences of the United States of America, 115, 8376–8381. Available from: 10.1073/pnas.1810430115 30061421 PMC6099890

[tpj70130-bib-0111] Liu, A. , Mair, A. , Matos, J.L. , Vollbrecht, M. , Xu, S.‐L. & Bergmann, D.C. (2024) bHLH transcription factors cooperate with chromatin remodelers to regulate cell fate decisions during Arabidopsis stomatal development. PLoS Biology, 22, e3002770. Available from: 10.1371/journal.pbio.3002770 39150946 PMC11357106

[tpj70130-bib-0112] Liu, B. , Li, C. , Li, X. , Wang, J. , Xie, W. , Woods, D.P. et al. (2024) The H3K4 demethylase JMJ1 is required for proper timing of flowering in *Brachypodium distachyon* . Plant Cell, 36, 2729–2745. Available from: 10.1093/plcell/koae124 38652680 PMC11218787

[tpj70130-bib-0113] Liu, H. , Yu, H. , Tang, G. & Huang, T. (2018) Small but powerful: function of microRNAs in plant development. Plant Cell Reports, 37, 515–528. Available from: 10.1007/s00299-017-2246-5 29318384

[tpj70130-bib-0114] Liu, H. , Zhang, H. , Dong, Y.X. , Hao, Y.J. & Zhang, X.S. (2018) DNA METHYLTRANSFERASE1‐mediated shoot regeneration is regulated by cytokinin‐induced cell cycle in Arabidopsis. The New Phytologist, 217, 219–232. Available from: 10.1111/nph.14814 28960381

[tpj70130-bib-0115] Loeffler, D. (2023) Editorial: single cell dynamics and cell cycle length variation. Frontiers in Cell and Development Biology, 11, 1321316. Available from: 10.3389/fcell.2023.1321316 PMC10628721 37941900

[tpj70130-bib-0116] Lotan, T. , Ohto, M. , Yee, K.M. , West, M.A. , Lo, R. , Kwong, R.W. et al. (1998) Arabidopsis LEAFY COTYLEDON1 is sufficient to induce embryo development in vegetative cells. Cell, 93, 1195–1205. Available from: 10.1016/s0092-8674(00)81463-4 9657152

[tpj70130-bib-0117] Lu, Q. , Shi, W. , Zhang, F. & Ding, Y. (2024) ATX1 and HUB1/2 promote recruitment of the transcription elongation factor VIP2 to modulate the floral transition in Arabidopsis. The Plant Journal: For Cell and Molecular Biology, 118, 1760–1773. Available from: 10.1111/tpj.16707 38446797

[tpj70130-bib-0118] Lumba, S. , Tsuchiya, Y. , Delmas, F. , Hezky, J. , Provart, N.J. , Shi Lu, Q. et al. (2012) The embryonic leaf identity gene FUSCA3 regulates vegetative phase transitions by negatively modulating ethylene‐regulated gene expression in Arabidopsis. BMC Biology, 10, 8. Available from: 10.1186/1741-7007-10-8 22348746 PMC3305478

[tpj70130-bib-0119] Lv, Z. , Zhao, W. , Kong, S. , Li, L. & Lin, S. (2023) Overview of molecular mechanisms of plant leaf development: a systematic review. Frontiers in Plant Science, 14, 1293424. Available from: 10.3389/fpls.2023.1293424 38146273 PMC10749370

[tpj70130-bib-0120] Lymperopoulos, P. , Msanne, J. & Rabara, R. (2018) Phytochrome and phytohormones: working in tandem for plant growth and development. Frontiers in Plant Science, 9, 1037. Available from: 10.3389/fpls.2018.01037 30100912 PMC6072860

[tpj70130-bib-0121] Ma, Y. , McKay, D.J. & Buttitta, L. (2019) Changes in chromatin accessibility ensure robust cell cycle exit in terminally differentiated cells. PLoS Biology, 17, e3000378. Available from: 10.1371/journal.pbio.3000378 31479438 PMC6743789

[tpj70130-bib-0122] MacAlister, C.A. , Ohashi‐Ito, K. & Bergmann, D.C. (2007) Transcription factor control of asymmetric cell divisions that establish the stomatal lineage. Nature, 445, 537–540. Available from: 10.1038/nature05491 17183265

[tpj70130-bib-0123] Majda, M. , Trozzi, N. , Mosca, G. & Smith, R.S. (2022) How cell geometry and cellular patterning influence tissue stiffness. International Journal of Molecular Sciences, 23, 5651. Available from: 10.3390/ijms23105651 35628463 PMC9145195

[tpj70130-bib-0124] Manuela, D. & Xu, M. (2020) Juvenile leaves or adult leaves: determinants for vegetative phase change in flowering plants. International Journal of Molecular Sciences, 21, 9753. Available from: 10.3390/ijms21249753 33371265 PMC7766579

[tpj70130-bib-0125] Maple, R. , Zhu, P. , Hepworth, J. , Wang, J.‐W. & Dean, C. (2024) Flowering time: from physiology, through genetics to mechanism. Plant Physiology, 195, 190–212. Available from: 10.1093/plphys/kiae109 38417841 PMC11060688

[tpj70130-bib-0126] Marhavý, P. , Montesinos, J.C. , Abuzeineh, A. , Van Damme, D. , Vermeer, J.E.M. , Duclercq, J. et al. (2016) Targeted cell elimination reveals an auxin‐guided biphasic mode of lateral root initiation. Genes & Development, 30, 471–483. Available from: 10.1101/gad.276964.115 26883363 PMC4762431

[tpj70130-bib-0127] Matsuda, M. , Hayashi, H. , Garcia‐Ojalvo, J. , Yoshioka‐Kobayashi, K. , Kageyama, R. , Yamanaka, Y. et al. (2020) Species‐specific segmentation clock periods are due to differential biochemical reaction speeds. Science, 369, 1450–1455. Available from: 10.1126/science.aba7668 32943519

[tpj70130-bib-0128] Meng, L.‐S. , Bao, Q.‐X. , Mu, X.‐R. , Tong, C. , Cao, X.‐Y. , Huang, J.‐J. et al. (2021) Glucose‐ and sucrose‐signaling modules regulate the Arabidopsis juvenile‐to‐adult phase transition. Cell Reports, 36, 109348. Available from: 10.1016/j.celrep.2021.109348 34260932

[tpj70130-bib-0129] Meyerowitz, E.M. (2002) Plants compared to animals: the broadest comparative study of development. Science, 295, 1482–1485. Available from: 10.1126/science.1066609 11859185

[tpj70130-bib-0130] Miryeganeh, M. , Yamaguchi, M. & Kudoh, H. (2018) Synchronisation of Arabidopsis flowering time and whole‐plant senescence in seasonal environments. Scientific Reports, 8, 10282. Available from: 10.1038/s41598-018-28580-x 29980723 PMC6035182

[tpj70130-bib-0131] Miyashima, S. , Koi, S. , Hashimoto, T. & Nakajima, K. (2011) Non‐cell‐autonomous microRNA165 acts in a dose‐dependent manner to regulate multiple differentiation status in the Arabidopsis root. Development (Cambridge, England), 138, 2303–2313. Available from: 10.1242/dev.060491 21558378

[tpj70130-bib-0132] Motte, H. , Vanneste, S. & Beeckman, T. (2019) Molecular and environmental regulation of root development. Annual Review of Plant Biology, 70, 465–488. Available from: 10.1146/annurev-arplant-050718-100423 30822115

[tpj70130-bib-0133] Muñoz, A. , Mangano, S. , González‐García, M.P. , Contreras, R. , Sauer, M. , De Rybel, B. et al. (2017) RIMA‐dependent nuclear accumulation of IYO triggers auxin‐irreversible cell differentiation in Arabidopsis. Plant Cell, 29, 575–588. Available from: 10.1105/tpc.16.00791 28223441 PMC5385956

[tpj70130-bib-0134] Nanda, A.K. , El Habti, A. , Hocart, C.H. & Masle, J. (2019) ERECTA receptor‐kinases play a key role in the appropriate timing of seed germination under changing salinity. Journal of Experimental Botany, 70, 6417–6435. Available from: 10.1093/jxb/erz385 31504732 PMC6859730

[tpj70130-bib-0135] Née, G. , Kramer, K. , Nakabayashi, K. , Yuan, B. , Xiang, Y. , Miatton, E. et al. (2017) DELAY OF GERMINATION1 requires PP2C phosphatases of the ABA signalling pathway to control seed dormancy. Nature Communications, 8, 72. Available from: 10.1038/s41467-017-00113-6 PMC5509711 28706187

[tpj70130-bib-0136] Nobori, T. , Oliva, M. , Lister, R. & Ecker, J.R. (2023) Multiplexed single‐cell 3D spatial gene expression analysis in plant tissue using PHYTOMap. Nature Plants, 9, 1026–1033. Available from: 10.1038/s41477-023-01439-4 37308583 PMC10356616

[tpj70130-bib-0137] Nolan, T.M. & Shahan, R. (2023) Resolving plant development in space and time with single‐cell genomics. Current Opinion in Plant Biology, 76, 102444. Available from: 10.1016/j.pbi.2023.102444 37696725

[tpj70130-bib-0138] Ogneva, Z.V. , Dubrovina, A.S. & Kiselev, K.V. (2016) Age‐associated alterations in DNA methylation and expression of methyltransferase and demethylase genes in *Arabidopsis thaliana* . Biologia Plantarum, 60, 628–634. Available from: 10.1007/s10535-016-0638-y

[tpj70130-bib-0139] Ohashi‐Ito, K. & Bergmann, D.C. (2006) Arabidopsis FAMA controls the final proliferation/differentiation switch during stomatal development. Plant Cell, 18, 2493–2505. Available from: 10.1105/tpc.106.046136 17088607 PMC1626605

[tpj70130-bib-0140] Oliva, M. & Lister, R. (2023) Exploring the identity of individual plant cells in space and time. The New Phytologist, 240, 61–67. Available from: 10.1111/nph.19153 37483019 PMC10952157

[tpj70130-bib-0141] Ó'Maoiléidigh, D.S. , Stewart, D. , Zheng, B. , Coupland, G. & Wellmer, F. (2018) Floral homeotic proteins modulate the genetic program for leaf development to suppress trichome formation in flowers. Development (Cambridge, England), 145, dev157784. Available from: 10.1242/dev.157784 29361563

[tpj70130-bib-0142] Pan, J. , Zhang, H. , Zhan, Z. , Zhao, T. & Jiang, D. (2023) A REF6‐dependent H3K27me3‐depleted state facilitates gene activation during germination in Arabidopsis. Journal of Genetics and Genomics = Yi Chuan Xue Bao, 50, 178–191. Available from: 10.1016/j.jgg.2022.09.001 36113770

[tpj70130-bib-0143] Pan, J. , Zhao, F. , Zhang, G. , Pan, Y. , Sun, L. , Bao, N. et al. (2019) Control of de novo root regeneration efficiency by developmental status of Arabidopsis leaf explants. Journal of Genetics and Genomics, 46, 133–140. Available from: 10.1016/j.jgg.2019.03.001 30928533

[tpj70130-bib-0144] Patil, G. & van Zon, J.S. (2024) Timers, variability, and body‐wide coordination: *C. elegans* as a model system for whole‐animal developmental timing. Current Opinion in Genetics & Development, 85, 102172. Available from: 10.1016/j.gde.2024.102172 38432125

[tpj70130-bib-0145] Petersen, M. , Ebstrup, E. & Rodriguez, E. (2024) Going through changes – the role of autophagy during reprogramming and differentiation. Journal of Cell Science, 137, jcs261655. Available from: 10.1242/jcs.261655 38393817

[tpj70130-bib-0146] Pillitteri, L.J. , Sloan, D.B. , Bogenschutz, N.L. & Torii, K.U. (2007) Termination of asymmetric cell division and differentiation of stomata. Nature, 445, 501–505. Available from: 10.1038/nature05467 17183267

[tpj70130-bib-0147] Poethig, R.S. & Fouracre, J. (2024) Temporal regulation of vegetative phase change in plants. Developmental Cell, 59, 4–19. Available from: 10.1016/j.devcel.2023.11.010 38194910 PMC10783531

[tpj70130-bib-0148] Pollington, H.Q. , Seroka, A.Q. & Doe, C.Q. (2023) From temporal patterning to neuronal connectivity in *Drosophila* type I neuroblast lineages. Seminars in Cell & Developmental Biology, 142, 4–12. Available from: 10.1016/j.semcdb.2022.05.022 35659165 PMC9938700

[tpj70130-bib-0149] Rahni, R. & Birnbaum, K.D. (2019) Week‐long imaging of cell divisions in the Arabidopsis root meristem. Plant Methods, 15, 30. Available from: 10.1186/s13007-019-0417-9 30988691 PMC6446972

[tpj70130-bib-0150] Rahul, P.V. , Yadukrishnan, P. , Sasidharan, A. & Datta, S. (2024) The B‐box protein BBX13/COL15 suppresses photoperiodic flowering by attenuating the action of CONSTANS in Arabidopsis. Plant, Cell & Environment, 47(12), pce.15120. Available from: 10.1111/pce.15120 39189944

[tpj70130-bib-0151] Raines, T. , Shanks, C. , Cheng, C.‐Y. , McPherson, D. , Argueso, C.T. , Kim, H.J. et al. (2016) The cytokinin response factors modulate root and shoot growth and promote leaf senescence in Arabidopsis. The Plant Journal: For Cell and Molecular Biology, 85, 134–147. Available from: 10.1111/tpj.13097 26662515

[tpj70130-bib-0152] Rayon, T. (2023) Cell time: how cells control developmental timetables. Science Advances, 9, eadh1849. Available from: 10.1126/sciadv.adh1849 36888707 PMC9995029

[tpj70130-bib-0153] Rayon, T. , Stamataki, D. , Perez‐Carrasco, R. , Garcia‐Perez, L. , Barrington, C. , Melchionda, M. et al. (2020) Species‐specific pace of development is associated with differences in protein stability. Science, 369, eaba7667. Available from: 10.1126/science.aba7667 32943498 PMC7116327

[tpj70130-bib-0154] Raz, V. , Bergervoet, J.H.W. & Koornneef, M. (2001) Sequential steps for developmental arrest in Arabidopsis seeds. Development, 128(2), 243–252. Available from: 10.1242/dev.128.2.243 11124119

[tpj70130-bib-0155] Reyes‐Hernández, B.J. & Maizel, A. (2023) Tunable recurrent priming of lateral roots in Arabidopsis: more than just a clock? Current Opinion in Plant Biology, 76, 102479. Available from: 10.1016/j.pbi.2023.102479 37857036

[tpj70130-bib-0156] Roeder, A.H.K. (2021) Arabidopsis sepals: a model system for the emergent process of morphogenesis. Quantitative Plant Biology, 2, e14. Available from: 10.1017/qpb.2021.12 36798428 PMC9931181

[tpj70130-bib-0157] Rohde, L.A. , Bercowsky‐Rama, A. , Valentin, G. , Naganathan, S.R. , Desai, R.A. , Strnad, P. et al. (2024) Cell‐autonomous timing drives the vertebrate segmentation clock's wave pattern. eLife, 13, RP93764. Available from: 10.7554/eLife.93764.2 39671306 PMC11643631

[tpj70130-bib-0158] Roszak, P. , Heo, J.‐O. , Blob, B. , Toyokura, K. , Sugiyama, Y. , de Luis Balaguer, M.A. et al. (2021) Cell‐by‐cell dissection of phloem development links a maturation gradient to cell specialization. Science, 374, eaba5531. Available from: 10.1126/science.aba5531 34941412 PMC8730638

[tpj70130-bib-0159] Rusnak, B. , Clark, F.K. , Vadde, B.V.L. & Roeder, A.H.K. (2024) What is a plant cell type in the age of single‐cell biology? It's complicated. Annual Review of Cell and Developmental Biology, 40, 301–328. Available from: 10.1146/annurev-cellbio-111323-102412 PMC12279474 38724025

[tpj70130-bib-0160] Ryu, K.H. , Zhu, Y. & Schiefelbein, J. (2021) Plant cell identity in the era of single‐cell transcriptomics. Annual Review of Genetics, 55, 479–496. Available from: 10.1146/annurev-genet-071719-020453 34530637

[tpj70130-bib-0161] Sajeev, N. , Koornneef, M. & Bentsink, L. (2024) A commitment for life: decades of unraveling the molecular mechanisms behind seed dormancy and germination. Plant Cell, 36, 1358–1376. Available from: 10.1093/plcell/koad328 38215009 PMC11062444

[tpj70130-bib-0162] Sakuraba, Y. (2021) Light‐mediated regulation of leaf senescence. International Journal of Molecular Sciences, 22, 3291. Available from: 10.3390/ijms22073291 33804852 PMC8037705

[tpj70130-bib-0163] Sakuraba, Y. , Jeong, J. , Kang, M.‐Y. , Kim, J. , Paek, N.‐C. & Choi, G. (2014) Phytochrome‐interacting transcription factors PIF4 and PIF5 induce leaf senescence in Arabidopsis. Nature Communications, 5, 4636. Available from: 10.1038/ncomms5636 25119965

[tpj70130-bib-0164] Sanagi, M. , Aoyama, S. , Kubo, A. , Lu, Y. , Sato, Y. , Ito, S. et al. (2021) Low nitrogen conditions accelerate flowering by modulating the phosphorylation state of FLOWERING BHLH 4 in Arabidopsis. Proceedings of the National Academy of Sciences, 118, e2022942118. Available from: 10.1073/pnas.2022942118 PMC8126780 33963081

[tpj70130-bib-0165] Sánchez, R. , Kim, M.Y. , Calonje, M. , Moon, Y.‐H. & Sung, Z.R. (2009) Temporal and spatial requirement of EMF1 activity for Arabidopsis vegetative and reproductive development. Molecular Plant, 2, 643–653. Available from: 10.1093/mp/ssp004 19825645

[tpj70130-bib-0166] Sanmartín, M. , Sauer, M. , Muñoz, A. & Rojo, E. (2012) MINIYO and transcriptional elongation: lifting the roadblock to differentiation. Transcription, 3, 25–28. Available from: 10.4161/trns.3.1.19303 22456317

[tpj70130-bib-0167] Sanmartín, M. , Sauer, M. , Muñoz, A. , Zouhar, J. , Ordóñez, A. , van de Ven, W.T.G. et al. (2011) A molecular switch for initiating cell differentiation in Arabidopsis. Current Biology: CB, 21, 999–1008. Available from: 10.1016/j.cub.2011.04.041 21620701

[tpj70130-bib-0168] Schoof, H. , Lenhard, M. , Haecker, A. , Mayer, K.F. , Jürgens, G. & Laux, T. (2000) The stem cell population of Arabidopsis shoot meristems in maintained by a regulatory loop between the CLAVATA and WUSCHEL genes. Cell, 100, 635–644. Available from: 10.1016/s0092-8674(00)80700-x 10761929

[tpj70130-bib-0169] Serivichyaswat, P.T. , Bartusch, K. , Leso, M. , Musseau, C. , Iwase, A. , Chen, Y. et al. (2022) High temperature perception in leaves promotes vascular regeneration and graft formation in distant tissues. Development (Cambridge, England), 149, dev200079. Available from: 10.1242/dev.200079 35217857 PMC8959136

[tpj70130-bib-0170] Shahan, R. , Hsu, C.‐W. , Nolan, T.M. , Cole, B.J. , Taylor, I.W. , Greenstreet, L. et al. (2022) A single‐cell Arabidopsis root atlas reveals developmental trajectories in wild‐type and cell identity mutants. Developmental Cell, 57, 543–560.e9. Available from: 10.1016/j.devcel.2022.01.008 35134336 PMC9014886

[tpj70130-bib-0171] Shahan, R. , Nolan, T.M. & Benfey, P.N. (2021) Single‐cell analysis of cell identity in the Arabidopsis root apical meristem: insights and opportunities. Journal of Experimental Botany, 72, 6679–6686. Available from: 10.1093/jxb/erab228 34018001 PMC8513161

[tpj70130-bib-0172] Shanmukhan, A.P. , Mathew, M.M. , Aiyaz, M. , Varaparambathu, V. , Kareem, A. , Radhakrishnan, D. et al. (2021) Regulation of touch‐stimulated de novo root regeneration from Arabidopsis leaves. Plant Physiology, 187, 52–58. Available from: 10.1093/plphys/kiab286 34618147 PMC8418404

[tpj70130-bib-0173] She, W. , Grimanelli, D. , Rutowicz, K. , Whitehead, M.W.J. , Puzio, M. , Kotlinski, M. et al. (2013) Chromatin reprogramming during the somatic‐to‐reproductive cell fate transition in plants. Development (Cambridge, England), 140, 4008–4019. Available from: 10.1242/dev.095034 24004947

[tpj70130-bib-0174] Shemer, O. , Landau, U. , Candela, H. , Zemach, A. & Eshed Williams, L. (2015) Competency for shoot regeneration from Arabidopsis root explants is regulated by DNA methylation. Plant Science: An International Journal of Experimental Plant Biology, 238, 251–261. Available from: 10.1016/j.plantsci.2015.06.015 26259192

[tpj70130-bib-0175] Skalák, J. , Vercruyssen, L. , Claeys, H. , Hradilová, J. , Černý, M. , Novák, O. et al. (2019) Multifaceted activity of cytokinin in leaf development shapes its size and structure in Arabidopsis. The Plant Journal: For Cell and Molecular Biology, 97, 805–824. Available from: 10.1111/tpj.14285 30748050

[tpj70130-bib-0176] Smit, M.E. & Bergmann, D.C. (2023) The stomatal fates: understanding initiation and enforcement of stomatal cell fate transitions. Current Opinion in Plant Biology, 76, 102449. Available from: 10.1016/j.pbi.2023.102449 37709566

[tpj70130-bib-0177] Smit, M.E. , Vatén, A. , Mair, A. , Northover, C.A.M. & Bergmann, D.C. (2023) Extensive embryonic patterning without cellular differentiation primes the plant epidermis for efficient post‐embryonic stomatal activities. Developmental Cell, 58, 506–521.e5. Available from: 10.1016/j.devcel.2023.02.014 36931268

[tpj70130-bib-0178] Somoza, S.C. , Sede, A.R. , Boccardo, N.A. & Muschietti, J.P. (2021) Keeping up with the RALFs: how these small peptides control pollen‐pistil interactions in Arabidopsis. The New Phytologist, 229, 14–18. Available from: 10.1111/nph.16817 32687662

[tpj70130-bib-0179] Song, Z. , Bian, Y. , Liu, J. , Sun, Y. & Xu, D. (2020) B‐box proteins: pivotal players in light‐mediated development in plants. Journal of Integrative Plant Biology, 62, 1293–1309. Available from: 10.1111/jipb.12935 32237198

[tpj70130-bib-0180] Soppe, W.J.J. & Bentsink, L. (2020) Seed dormancy back on track; its definition and regulation by DOG1. The New Phytologist, 228, 816–819. Available from: 10.1111/nph.16592 32267972 PMC7586819

[tpj70130-bib-0181] Soufi, A. & Dalton, S. (2016) Cycling through developmental decisions: how cell cycle dynamics control pluripotency, differentiation and reprogramming. Development (Cambridge, England), 143, 4301–4311. Available from: 10.1242/dev.142075 27899507 PMC5201050

[tpj70130-bib-0182] Suh, S.‐S. , Choi, K.‐R. & Lee, I. (2003) Revisiting phase transition during flowering in Arabidopsis. Plant & Cell Physiology, 44, 836–843. Available from: 10.1093/pcp/pcg109 12941876

[tpj70130-bib-0183] Suzuki, M. , Ketterling, M.G. , Li, Q.‐B. & McCarty, D.R. (2003) Viviparous1 alters global gene expression patterns through regulation of abscisic acid signaling. Plant Physiology, 132, 1664–1677. Available from: 10.1104/pp.103.022475 12857845 PMC167103

[tpj70130-bib-0184] Swiezewski, S. , Liu, F. , Magusin, A. & Dean, C. (2009) Cold‐induced silencing by long antisense transcripts of an Arabidopsis polycomb target. Nature, 462, 799–802. Available from: 10.1038/nature08618 20010688

[tpj70130-bib-0185] Swift, J. , Greenham, K. , Ecker, J.R. , Coruzzi, G.M. & Robertson McClung, C. (2022) The biology of time: dynamic responses of cell types to developmental, circadian and environmental cues. The Plant Journal: For Cell and Molecular Biology, 109, 764–778. Available from: 10.1111/tpj.15589 34797944 PMC9215356

[tpj70130-bib-0186] Tao, Z. , Shen, L. , Gu, X. , Wang, Y. , Yu, H. & He, Y. (2017) Embryonic epigenetic reprogramming by a pioneer transcription factor in plants. Nature, 551, 124–128. Available from: 10.1038/nature24300 29072296

[tpj70130-bib-0187] Tian, T. , Ma, L. , Liu, Y. , Xu, D. , Chen, Q. & Li, G. (2020) Arabidopsis FAR‐RED ELONGATED HYPOCOTYL3 integrates age and light signals to negatively regulate leaf senescence. Plant Cell, 32, 1574–1588. Available from: 10.1105/tpc.20.00021 32152188 PMC7203920

[tpj70130-bib-0188] Tsukagoshi, H. , Busch, W. & Benfey, P.N. (2010) Transcriptional regulation of ROS controls transition from proliferation to differentiation in the root. Cell, 143, 606–616. Available from: 10.1016/j.cell.2010.10.020 21074051

[tpj70130-bib-0189] van den Berg, C. , Willemsen, V. , Hage, W. , Weisbeek, P. & Scheres, B. (1995) Cell fate in the Arabidopsis root meristem determined by directional signalling. Nature, 378, 62–65. Available from: 10.1038/378062a0 7477287

[tpj70130-bib-0190] Van Den Broeck, L. , Gordon, M. , Inzé, D. , Williams, C. & Sozzani, R. (2020) Gene regulatory network inference: connecting plant biology and mathematical modeling. Frontiers in Genetics, 11, 457. Available from: 10.3389/fgene.2020.00457 32547596 PMC7270862

[tpj70130-bib-0191] Varapparambath, V. , Mathew, M.M. , Shanmukhan, A.P. , Radhakrishnan, D. , Kareem, A. , Verma, S. et al. (2022) Mechanical conflict caused by a cell‐wall‐loosening enzyme activates de novo shoot regeneration. Developmental Cell, 57, 2063–2080.e10. Available from: 10.1016/j.devcel.2022.07.017 36002002

[tpj70130-bib-0192] Vatov, E. , Zentgraf, U. & Ludewig, U. (2022) Moderate DNA methylation changes associated with nitrogen remobilization and leaf senescence in Arabidopsis. Journal of Experimental Botany, 73, 4733–4752. Available from: 10.1093/jxb/erac167 35552412 PMC9366325

[tpj70130-bib-0193] Verbelen, J.‐P. , De Cnodder, T. , Le, J. , Vissenberg, K. & Baluska, F. (2006) The root apex of *Arabidopsis thaliana* consists of four distinct zones of growth activities: meristematic zone, transition zone, fast elongation zone and growth terminating zone. Plant Signaling & Behavior, 1, 296–304. Available from: 10.4161/psb.1.6.3511 19517000 PMC2634244

[tpj70130-bib-0194] Voss, S.R. , Ponomareva, L.V. , Dwaraka, V.B. , Pardue, K.E. , Baddar, N.W.A.H. , Rodgers, A.K. et al. (2019) HDAC regulates transcription at the outset of axolotl tail regeneration. Scientific Reports, 9, 6751. Available from: 10.1038/s41598-019-43230-6 31043677 PMC6494824

[tpj70130-bib-0195] Wang, F. & Perry, S.E. (2013) Identification of direct targets of FUSCA3, a key regulator of Arabidopsis seed development. Plant Physiology, 161, 1251–1264. Available from: 10.1104/pp.112.212282 23314941 PMC3585594

[tpj70130-bib-0196] Wang, M.‐H. , Hsu, C.‐L. , Wu, C.‐H. , Chiou, L.‐L. , Tsai, Y.‐T. , Lee, H.‐S. et al. (2021) Timing does matter: nerve‐mediated HDAC1 paces the temporal expression of morphogenic genes during axolotl limb regeneration. Frontiers in Cell and Development Biology, 9, 641987. Available from: 10.3389/fcell.2021.641987 PMC8143519 34041236

[tpj70130-bib-0197] Wang, M.‐H. , Wu, C.‐H. , Huang, T.‐Y. , Sung, H.‐W. , Chiou, L.‐L. , Lin, S.‐P. et al. (2019) Nerve‐mediated expression of histone deacetylases regulates limb regeneration in axolotls. Developmental Biology, 449, 122–131. Available from: 10.1016/j.ydbio.2019.02.011 30826398

[tpj70130-bib-0198] Wang, Q. , Liu, M. , Quan, S. , Shi, Q. , Tian, T. , Zhang, H. et al. (2023) FAR‐RED ELONGATED HYPOCOTYL3 increases leaf longevity by delaying senescence in arabidopsis. Plant, Cell & Environment, 46, 1582–1595. Available from: 10.1111/pce.14554 36721872

[tpj70130-bib-0199] Wang, W. , Ryu, K.H. , Bruex, A. , Barron, C. & Schiefelbein, J. (2020) Molecular basis for a cell fate switch in response to impaired ribosome biogenesis in the Arabidopsis root epidermis. Plant Cell, 32, 2402–2423. Available from: 10.1105/tpc.19.00773 32371546 PMC7346552

[tpj70130-bib-0200] Ware, A. , Walker, C.H. , Šimura, J. , González‐Suárez, P. , Ljung, K. , Bishopp, A. et al. (2020) Auxin export from proximal fruits drives arrest in temporally competent inflorescences. Nature Plants, 6, 699–707. Available from: 10.1038/s41477-020-0661-z 32451444

[tpj70130-bib-0201] Weigel, D. , Alvarez, J. , Smyth, D.R. , Yanofsky, M.F. & Meyerowitz, E.M. (1992) LEAFY controls floral meristem identity in Arabidopsis. Cell, 69, 843–859. Available from: 10.1016/0092-8674(92)90295-n 1350515

[tpj70130-bib-0202] Weigel, D. & Nilsson, O. (1995) A developmental switch sufficient for flower initiation in diverse plants. Nature, 377, 495–500. 7566146 10.1038/377495a0

[tpj70130-bib-0203] Williams, B.P. , Bechen, L.L. , Pohlmann, D.A. & Gehring, M. (2022) Somatic DNA demethylation generates tissue‐specific methylation states and impacts flowering time. Plant Cell, 34, 1189–1206. Available from: 10.1093/plcell/koab319 34954804 PMC8972289

[tpj70130-bib-0204] Willoughby, A.C. & Nimchuk, Z.L. (2021) WOX going on: CLE peptides in plant development. Current Opinion in Plant Biology, 63, 102056. Available from: 10.1016/j.pbi.2021.102056 34077886 PMC8545713

[tpj70130-bib-0205] Wolf, S. (2022) Cell Wall signaling in plant development and defense. Annual Review of Plant Biology, 73, 323–353. Available from: 10.1146/annurev-arplant-102820-095312 35167757

[tpj70130-bib-0206] Woo, H.R. , Kim, H.J. , Nam, H.G. & Lim, P.O. (2013) Plant leaf senescence and death ‐ regulation by multiple layers of control and implications for aging in general. Journal of Cell Science, 126, 4823–4833. Available from: 10.1242/jcs.109116 24144694

[tpj70130-bib-0207] Wu, G. , Park, M.Y. , Conway, S.R. , Wang, J.‐W. , Weigel, D. & Poethig, R.S. (2009) The sequential action of miR156 and miR172 regulates developmental timing in Arabidopsis. Cell, 138, 750–759. Available from: 10.1016/j.cell.2009.06.031 19703400 PMC2732587

[tpj70130-bib-0208] Wu, G. & Poethig, R.S. (2006) Temporal regulation of shoot development in *Arabidopsis thaliana* by miR156 and its target SPL3. Development (Cambridge, England), 133, 3539–3547. Available from: 10.1242/dev.02521 16914499 PMC1610107

[tpj70130-bib-0209] Wu, W. , Du, K. , Kang, X. & Wei, H. (2021) The diverse roles of cytokinins in regulating leaf development. Horticulture Research, 8, 118. Available from: 10.1038/s41438-021-00558-3 34059666 PMC8167137

[tpj70130-bib-0210] Xiao, J. , Jin, R. & Wagner, D. (2017) Developmental transitions: integrating environmental cues with hormonal signaling in the chromatin landscape in plants. Genome Biology, 18, 88. Available from: 10.1186/s13059-017-1228-9 28490341 PMC5425979

[tpj70130-bib-0211] Yamaguchi, N. (2021) LEAFY, a Pioneer transcription factor in plants: a mini‐review. Frontiers in Plant Science, 12, 701406. Available from: 10.3389/fpls.2021.701406 34290727 PMC8287900

[tpj70130-bib-0212] Yan, A. & Chen, Z. (2020) The control of seed dormancy and germination by temperature, light and nitrate. The Botanical Review, 86, 39–75. Available from: 10.1007/s12229-020-09220-4

[tpj70130-bib-0213] Yang, X. , Tong, A. , Yan, B. & Wang, X. (2017) Governing the silencing state of chromatin: the roles of Polycomb repressive complex 1 in Arabidopsis. Plant & Cell Physiology, 58, 198–206. Available from: 10.1093/pcp/pcw209 28069891

[tpj70130-bib-0214] Ye, R. , Wang, M. , Du, H. , Chhajed, S. , Koh, J. , Liu, K.‐H. et al. (2022) Glucose‐driven TOR‐FIE‐PRC2 signalling controls plant development. Nature, 609, 986–993. Available from: 10.1038/s41586-022-05171-5 36104568 PMC9530021

[tpj70130-bib-0215] Yoshida, N. , Yanai, Y. , Chen, L. , Kato, Y. , Hiratsuka, J. , Miwa, T. et al. (2001) EMBRYONIC FLOWER2, a novel polycomb group protein homolog, mediates shoot development and flowering in Arabidopsis. Plant Cell, 13, 2471–2481. Available from: 10.1105/tpc.010227 11701882 PMC139465

[tpj70130-bib-0216] Zhang, A. , Matsuoka, K. , Kareem, A. , Robert, M. , Roszak, P. , Blob, B. et al. (2022) Cell‐wall damage activates DOF transcription factors to promote wound healing and tissue regeneration in *Arabidopsis thaliana* . Current Biology: CB, 32, 1883–1894. Available from: 10.1016/j.cub.2022.02.069 35320706

[tpj70130-bib-0217] Zhang, K. , Diederich, L. & John, P.C.L. (2005) The cytokinin requirement for cell division in cultured *Nicotiana plumbaginifolia* cells can be satisfied by yeast Cdc25 protein tyrosine phosphatase: implications for mechanisms of cytokinin response and plant development. Plant Physiology, 137, 308–316. Available from: 10.1104/pp.104.051938 15618425 PMC548861

[tpj70130-bib-0218] Zhang, T.‐Q. , Lian, H. , Tang, H. , Dolezal, K. , Zhou, C.‐M. , Yu, S. et al. (2015) An intrinsic microRNA timer regulates progressive decline in shoot regenerative capacity in plants. Plant Cell, 27, 349–360. Available from: 10.1105/tpc.114.135186 25649435 PMC4456919

[tpj70130-bib-0219] Zhang, Y.‐M. , Guo, P. , Xia, X. , Guo, H. & Li, Z. (2021) Multiple layers of regulation on leaf senescence: new advances and perspectives. Frontiers in Plant Science, 12, 788996. Available from: 10.3389/fpls.2021.788996 34938309 PMC8685244

[tpj70130-bib-0220] Zhang, Z. , Luo, X. , Yang, Y. & He, Y. (2023) Cold induction of nuclear FRIGIDA condensation in Arabidopsis. Nature, 619, E27–E32. Available from: 10.1038/s41586-023-06189-z 37438599 PMC10338335

[tpj70130-bib-0221] Zheng, C. , Ye, M. , Sang, M. & Wu, R. (2019) A regulatory network for miR156‐SPL module in *Arabidopsis thaliana* . International Journal of Molecular Sciences, 20, 6166. Available from: 10.3390/ijms20246166 31817723 PMC6940959

[tpj70130-bib-0222] Zheng, J. , Chen, F. , Wang, Z. , Cao, H. , Li, X. , Deng, X. et al. (2012) A novel role for histone methyltransferase KYP/SUVH4 in the control of Arabidopsis primary seed dormancy. The New Phytologist, 193, 605–616. Available from: 10.1111/j.1469-8137.2011.03969.x 22122546

[tpj70130-bib-0223] Zheng, L. , Otani, M. , Kanno, Y. , Seo, M. , Yoshitake, Y. , Yoshimoto, K. et al. (2022) Seed dormancy 4 like1 of Arabidopsis is a key regulator of phase transition from embryo to vegetative development. The Plant Journal, 112, 460–475. Available from: 10.1111/tpj.15959 36036886

[tpj70130-bib-0224] Zhong, S. , Li, L. , Wang, Z. , Ge, Z. , Li, Q. , Bleckmann, A. et al. (2022) RALF peptide signaling controls the polytubey block in Arabidopsis. Science, 375, 290–296. Available from: 10.1126/science.abl4683 35050671 PMC9040003

[tpj70130-bib-0225] Zhou, Y. , Honda, M. , Zhu, H. , Zhang, Z. , Guo, X. , Li, T. et al. (2015) Spatiotemporal sequestration of miR165/166 by Arabidopsis argonaute10 promotes shoot apical meristem maintenance. Cell Reports, 10, 1819–1827. Available from: 10.1016/j.celrep.2015.02.047 25801022

[tpj70130-bib-0226] Zhu, M. , Chen, W. , Mirabet, V. , Hong, L. , Bovio, S. , Strauss, S. et al. (2020) Robust organ size requires robust timing of initiation orchestrated by focused auxin and cytokinin signalling. Nature Plants, 6, 686–698. Available from: 10.1038/s41477-020-0666-7 32451448 PMC7299778

[tpj70130-bib-0227] Zhu, P. , Lister, C. & Dean, C. (2021) Cold‐induced Arabidopsis FRIGIDA nuclear condensates for FLC repression. Nature, 599, 657–661. Available from: 10.57760/SCIENCEDB.01119 34732891 PMC8612926

[tpj70130-bib-0228] Zhu, Y. , Klasfeld, S. , Jeong, C.W. , Jin, R. , Goto, K. , Yamaguchi, N. et al. (2020) TERMINAL FLOWER 1‐FD complex target genes and competition with FLOWERING LOCUS T. Nature Communications, 11, 5118. Available from: 10.1038/s41467-020-18782-1 PMC7550357 33046692

[tpj70130-bib-0229] Zuch, D.T. , Herrmann, A. , Kim, E.‐D. & Torii, K.U. (2023) Cell cycle dynamics during stomatal development: window of MUTE action and ramification of its loss‐of‐function on an uncommitted precursor. Plant & Cell Physiology, 64, 325–335. Available from: 10.1093/pcp/pcad002 36609867 PMC10016323

